# Urolithin A promotes p62-dependent lysophagy to prevent acute retinal neurodegeneration

**DOI:** 10.1186/s13024-024-00739-3

**Published:** 2024-06-18

**Authors:** Juan Ignacio Jiménez-Loygorri, Álvaro Viedma-Poyatos, Raquel Gómez-Sintes, Patricia Boya

**Affiliations:** 1 Department of Cellular and Molecular Biology, Centro de Investigaciones Biológicas Margarita Salas, CSIC, Madrid, Spain; 2 Department of Neuroscience and Movement Science, Faculty of Science and Medicine, University of Fribourg, Fribourg, Switzerland

**Keywords:** Age-related macular degeneration, Sodium iodate, Urolithin A, Autophagy, Lysophagy, Lysosomal membrane permeabilization, SQSTM1/p62

## Abstract

**Background:**

Age-related macular degeneration (AMD) is the leading cause of blindness in elderly people in the developed world, and the number of people affected is expected to almost double by 2040. The retina presents one of the highest metabolic demands in our bodies that is partially or fully fulfilled by mitochondria in the neuroretina and retinal pigment epithelium (RPE), respectively. Together with its post-mitotic status and constant photooxidative damage from incoming light, the retina requires a tightly-regulated housekeeping system that involves autophagy. The natural polyphenol Urolithin A (UA) has shown neuroprotective benefits in several models of aging and age-associated disorders, mostly attributed to its ability to induce mitophagy and mitochondrial biogenesis. Sodium iodate (SI) administration recapitulates the late stages of AMD, including geographic atrophy and photoreceptor cell death.

**Methods:**

A combination of in vitro, ex vivo and in vivo models were used to test the neuroprotective potential of UA in the SI model. Functional assays (OCT, ERGs), cellular analysis (flow cytometry, qPCR) and fine confocal microscopy (immunohistochemistry, tandem selective autophagy reporters) helped address this question.

**Results:**

UA alleviated neurodegeneration and preserved visual function in SI-treated mice. Simultaneously, we observed severe proteostasis defects upon SI damage induction, including autophagosome accumulation, that were resolved in animals that received UA. Treatment with UA restored autophagic flux and triggered PINK1/Parkin-dependent mitophagy, as previously reported in the literature. Autophagy blockage caused by SI was caused by severe lysosomal membrane permeabilization. While UA did not induce lysosomal biogenesis, it did restore upcycling of permeabilized lysosomes through lysophagy. Knockdown of the lysophagy adaptor SQSTM1/p62 abrogated viability rescue by UA in SI-treated cells, exacerbated lysosomal defects and inhibited lysophagy.

**Conclusions:**

Collectively, these data highlight a novel putative application of UA in the treatment of AMD whereby it bypasses lysosomal defects by promoting p62-dependent lysophagy to sustain proteostasis.

**Graphical Abstract:**

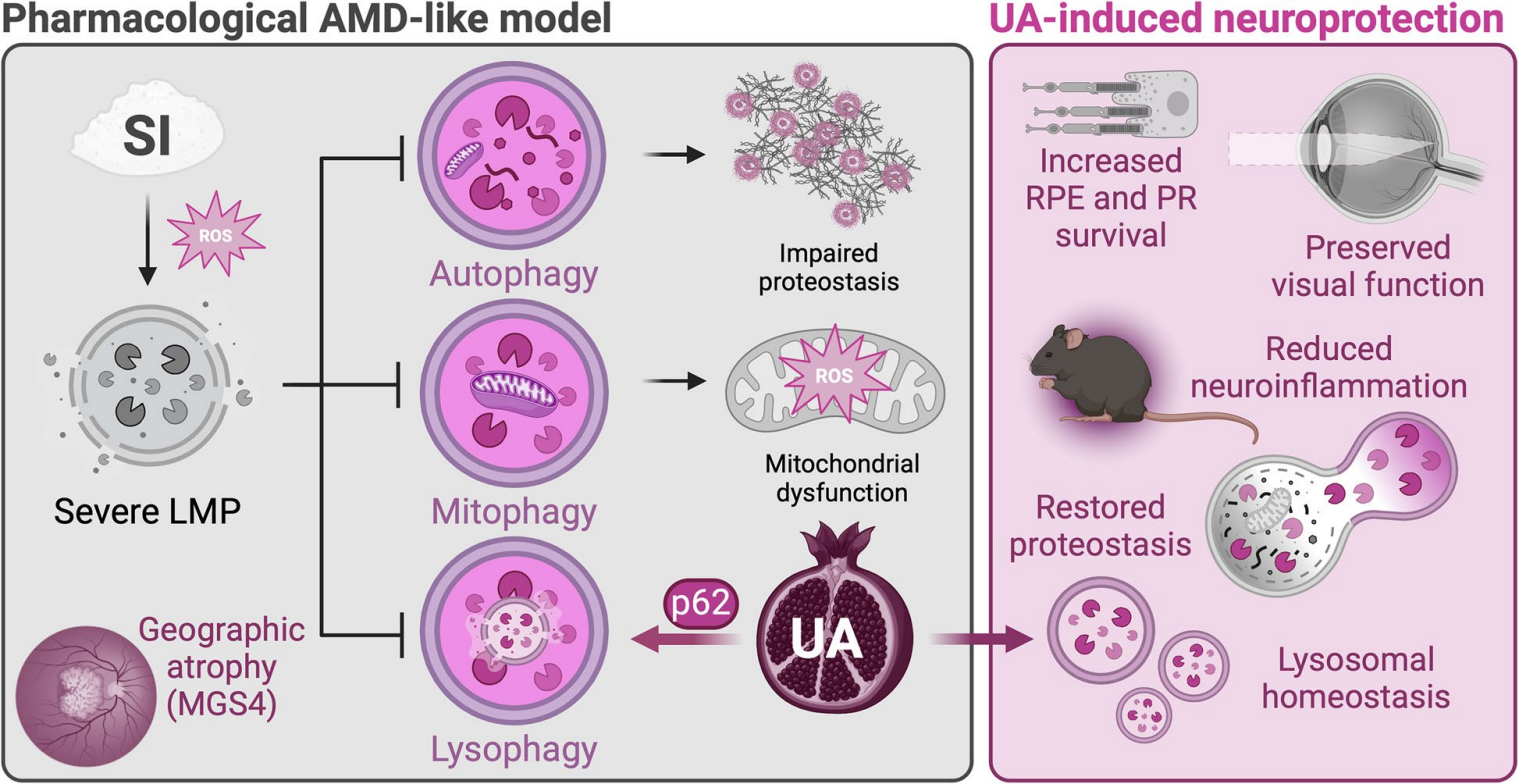

**Supplementary Information:**

The online version contains supplementary material available at 10.1186/s13024-024-00739-3.

## Background

Age-related macular degeneration (AMD) is the most prevalent ocular disease in the elder population and can lead to severe and permanent loss of central vision. Several environmental risk factors, such as smoking or hypertension, and genetic factors, like the CFH^Y402H^ polymorphism, have been linked to a higher AMD incidence, but aging remains the main risk factor. Currently, it has been estimated that around 200 million people worldwide are affected by AMD [1], and this number is projected to double by 2040 due to population aging [2]. This disease is characterized by progressive bilateral degeneration of the macula, a region located in the central part of the retina and enriched in cone photoreceptors that allows for high-resolution, color vision. One of the main caveats is the late diagnosis of the disease, usually when the first signs of central vision loss are detected by the patient and it can be classified as “dry” or “wet” AMD. Wet AMD involves choroidal neovascularization that disrupts the RPE leading to neuroretina invasion, edema, local inflammation and, eventually, photoreceptor cell death. Even though it only accounts for 10% of AMD diagnoses, it can be monitored and treated using anti-angiogenic immunotherapy (anti-VEGF). Dry AMD represents 90% of cases and its etiology is not fully understood, but includes a progressive deposition of extracellular debris (including lipids, oxidized proteins, apolipoproteins, complement components, trace elements) between the RPE and Bruch’s membrane, termed drusen. In a similar fashion to wet AMD, drusen eventually disrupt the RPE monolayer (geographic atrophy) and trigger cell death of adjacent photoreceptors [2].

Macroautophagy is the main intracellular catabolic pathway and is in charge of recycling damaged or superfluous components. The cargo to be degraded is recognized via diverse mechanisms, such as ubiquitination or direct binding of specific lipid species to autophagosome-associated LC3, and engulfed inside a double-membrane vesicle called autophagosome. Autophagosomes will eventually fuse with lysosomes where the cargo will be degraded to its minimum components (amino acids, simple lipids, ions) by acidic hydrolases [3]. In the past couple decades, different selective autophagy pathways targeting specific substrates have been described, including mitophagy (mitochondria), lipophagy (lipid droplets) or lysophagy (lysosomes) [4]. Studies using primary RPE cells derived from AMD patients point to deficient autophagy, e.g. accumulation of LC3-II [5] or abnormal lysosome morphology [6], and dysfunctional mitochondria [7] as drivers of AMD etiopathogenesis.

Lysosomes within the RPE have to deal with the highest phagocytic load in the human body and eliminate all the resulting exogenous debris from performing the visual cycle, a process whereby the RPE internalizes and replenishes the chromophore from photoreceptors [8]. On top of that, its post-mitotic status renders imperative a tight regulation of autophagy to sustain proteostasis and recycle intracellular and extracellular debris throughout a lifetime. Indeed, RPE-specific autophagy-deficient mice, such as *Atg5*^*ΔRPE*^ or *Atg7*^*ΔRPE*^, present severe alterations that lead to an AMD-like phenotype [9] and loss of visual function [10], even though photoreceptors and other retinal neurons still maintain functional autophagy.

Recently, our group reported that *Ambra1*^+*/gt*^ mice, which display just slightly reduced autophagic flux, present accelerated RPE and neuroretina aging as well as increased susceptibility to the sodium iodate pharmacological model of geographic atrophy [11]. Mice lacking the lysosome-associated protein LAMP-2 similarly present RPE alterations reminiscent of those found in dry AMD patients [12]. Here, we report that sodium iodate triggers macroautophagy, mitophagy and lysophagy but, ultimately, causes lysosomal membrane permeabilization leading to blockage of autophagic degradation. Treatment with the natural polyphenol Urolithin A prevented loss of visual function by promoting p62-dependent lysophagy to eliminate damaged lysosomes.

## Methods

### Animal models

All in vivo experiments using mice were performed according to EU guidelines and the ARVO Statement for the Use of Animals in Ophthalmic and Vision Research, and were authorized by the institutional bioethics committee and Comunidad de Madrid (PROEX 154.3/21). C57BL/6J wild-type mice were obtained from The Jackson Laboratory (Bar Harbor, USA). C57BL/6J *mito*-QC mitophagy reporter mice were generated and kindly gifted by Prof. Ian Ganley (University of Dundee, Scotland) [13]. *mito*-QC colony was genotyped by end-point PCR to ensure homozygosity using the following primers: set 1, 5′-CAAAGACCCCAACGAGAAGC-3′ and 5′-CCCAAGGCACACAAAAAACC-3′; set 2, 5′-CTCTTCCCTCGTGATCTGCAACTCC-3′ and 5′-CATGTCTTTAATCTACCTCGATGG-3′. Mice were bred and housed at the CIB-CSIC animal facility, on a 12/12 h light/dark cycle with *ad libitum* access to food (standard chow) and water. Males and females were equally distributed amongst all groups and young mice (2–3 months) were used for all experiments. To induce retinal degeneration, mice were intraperitoneally injected once with 50 mg/kg sodium iodate (NaIO3, SI; S4007, Merck) and sacrificed after seven days, control group received a single saline injection. 2.3 mg/kg Urolithin A (UA; 6762, Tocris) was intraperitoneally injected daily for the indicated time windows and control group received a vehicle (2.5% DMSO) injection daily. One week of 50 mg/kg SI treatment was selected based on preliminary experiments showing uniform and significant degeneration across the nasotemporal axis (data not shown). Animals were sacrificed by cervical dislocation between 9:00–10:00 a.m. to avoid circadian variations in autophagy, metabolism and the visual cycle.

### Organotypic eyecup culture

For ex vivo eyecup culture, eyes were enucleated and rinsed in sterile PBS. A circular incision was made along the limbus and the cornea and lens were carefully removed. The optic nerve was sectioned and any unwanted tissue was removed. Posterior eyecups were maintained in a humidified incubator at 37ºC and 5% CO_2_ inside a 24-well plate with Neurobasal™ Medium (21,103,049, Gibco) containing 2% B-27 (17,504,044, Gibco), 1% N-2 (17,502,001, Gibco), 1% penicillin/streptomycin (15,140,122, Gibco) and 800 μM L-Glutamine (25,030,081, Gibco). SI and/or UA were added and were cultured for 6 h. Whole eyecups were fixed using 4% PFA (50–980-487, EMS) for 30 min on ice. Neuroretina and RPE were isolated and fixed for an additional 90 min, then washed with PBS and processed for flat mount immunohistochemistry.

### ARPE-19 cell line culture

ARPE-19 cells (ATCC, CRL-2302) were maintained in 1:1 high-glucose DMEM (41,966–029, Gibco) and F12 (21,765,037, Gibco) supplemented with 15% FBS (F7524, Merck), 2 mM L-glutamine, and 1 U/mL Pen/Strep antibiotics, and stored in a humidified incubator at 37ºC, 5% CO_2_. Cells stably expressing the *mito*-QC (mitophagy) or MAP (macroautophagy) reporters were generated in the laboratory of Prof. Ian Ganley (University of Dundee, Scotland) and selected using 800 μg/mL Hygromycin B (10,453,982, Gibco). For immunofluorescence and flow cytometry analysis, 5 × 10^4^ cells were seeded in a 24-well plate. For RT-qPCR analysis, 1 × 10^5^ cells were seeded in 12-well plates. Whenever indicated, cells were treated with 20 mM SI, 100 μM UA, 50 nM Bafilomycin A1 (B1793, Merck), EBSS (starvation; E2888, Merck), 1 mM deferiprone (DFP; 379,409, Merck), 25 μM CCCP (C2759, Merck) or 1 mM LLOMe (L7393, Merck) for the indicated timepoints. Treatment with 20 mM SI was selected based on preliminary dose- and time-response studies performed to find a concentration that did not induce cell death at earlier timepoints (6–12 h) yet achieved 40–50% cell death by 24 h (data not shown).

### Fluorescent reporter plasmid transfection

For reporter fluorescence assays, 2.5 × 10^4^ ARPE-19 cells were seeded in a 24-well plate containing coverslips and transfected overnight with 1 μg/mL of the indicated plasmids (Supplementary Table 2) using Lipofectamine 3000 (L300015, Thermo Fisher) following manufacturer’s instructions. Treatments were added for the indicated timepoints and cells were fixed for 15 min using 3.7% PFA containing 175 mM HEPES pH 7.0. To generate a stable lysophagy reporter ARPE-19 cell line, 2 × 10^5^ cells were seeded in a 60 mm culture plate and grown until 70–80% confluence. Cells were transfected overnight with 1 μg/mL ptf-Galectin3 (64,149, Addgene). Medium was replaced and the positive population was selected using 1 mg/mL Geneticin/G418 (11,811–023, Gibco) during an additional 24 h. To obtain a pool with homogeneous reporter expression, GFP^high^RFP^high^ cells were sorted using a FACSAria Fusion Cell Sorter (Beckton-Dickinson). Cells were expanded and passaged before use in downstream experiments.

### siRNA-mediated gene knockdown and validation

Gene knockdown was achieved by transfecting the cells for 24 h with Silencer Select Pre-Designed siRNAs (PINK1, s35166; PARK2, s530998; UBE2QL1, s43825; SQSTM1, s16961; ATG5, s18160; Thermo Fisher) and Lipofectamine RNAiMAX (13,778,075, Thermo Fisher) following manufacturer’s instructions. Knockdown efficiency was validated by RT-qPCR.

### Electroretinography and optical coherence tomography (OCT)

Mice were dark-adapted overnight and all manipulations were performed under low-intensity red light. The animals were anesthetized by intraperitoneal injection (50 mg/kg ketamine, 0.3 mg/kg medetomidine) and both pupils were dilated using 1% tropicamide. During the procedure, mice were placed on top of a heating pad at 37ºC. A drop of 2% methocel (Omnivision GmbH) was added to each eye and corneal electrodes were carefully positioned parallel to the cornea. Reference electrode was placed in the mouth and ground electrode on the tail. Flash-induced ERG responses were recorded in response to increasing light stimuli produced by a calibrated Ganzfeld stimulator, and 5–20 readings were averaged for each intensity. Scotopic (0.00001, 0.0001, 0.001, 0.01 cd·s·m^−2^) and mixed (0.1, 0.32, 1, 3.2, 10, 32 cd·s·m^−2^) responses were recorded in dark conditions. Mice were light-adapted for 5 min and photopic responses (0.1, 0.32, 1, 3.2, 10, 32 cd·s·m^−2^) and flicker (32 cd·s·m^−2^) were recorded. ERG signals were amplified and band-filtered between 0.3 and 1000 Hz (CP511 AC amplifier; Grass Instruments). Electrical signals were digitized at 20 kHz with a power laboratory data acquisition board (AD Instruments). Wave amplitude was measured using LabChart 7 (AD Instruments). After ERG analysis, OCT recording was performed using a SPECTRALIS ophthalmic imaging platform (Heidelberg Engineering). For ERG and OCT assessment both eyes were analyzed and no major differences were detected between left and right eye, results show the average of both eyes for each mouse.

### Immunohistochemistry and immunocytochemistry

Eyes were enucleated, extraneous tissue dissected and fixed in 4% PFA for 2 h at RT. Whole eyes were cryoprotected using a gradient up to 30% sucrose (1.07651.1000, Millipore) and embedded in optimal cutting temperature (OCT) compound (4583, Sakura). Cryosections (12 µm-thick) were obtained using a Leica CM1950 cryostat and stored at -20ºC. Cryosections were obtained permeabilized by immersion for 15 min in 0.3% Triton X-100 in PBS pH 7.0 (T9284, Merck) and blocked for 1 h with block/perm buffer (10% NGS (G9023, Merck), 0.1% Triton X-100 in PBS). Similarly, neuroretina flat mounts were permeabilized with 2% Triton X-100 in PBS for 90 min and RPE/choroid flat mounts with 0.2% Triton X-100 in PBS for 1 h, then both blocked for 1 h with block/perm buffer. Samples were incubated overnight at 4ºC with primary antibodies diluted at the indicated concentration (Supplementary Table 1) in block/perm buffer. The following day, samples were incubated for 1 h at room temperature with fluorophore-conjugated secondary antibodies diluted 1:200 in block/perm buffer (Supplementary Table 1). Nuclei were counterstained with 1 µg/mL DAPI (D9542, Merck) for 15 min. Sections were washed 3 times with PBS after each step. Samples were mounted using Fluoromount-G (100–01, Bionova) and imaged with a 0.5–1 µm z-step using a Leica TCS SP8 confocal microscope equipped with a 63 × immersion objective. Immunocytochemistry was performed using BGT solution (3% BSA (MB04602, NZYtech), 0.25% Triton X-100 in PBS) and both primary and secondary antibodies (Supplementary Table 1) were incubated for 1 h at room temperature. Samples were mounted using ProLong Diamond (P36961, Thermo Fisher) and imaged as previously described for immunohistochemistry.

#### TUNEL assay

According to manufacturer instructions, apoptotic cells were detected using dUTP nick end labelling (TUNEL) with DeadEnd™ Fluorometric TUNEL System (G3250; Promega) or Click-iT™ TUNEL Alexa Fluor™ 647 Imaging Assay (C10247; Invitrogen) after primary antibody incubation.

#### Protein aggregate detection

According to manufacturer instructions, protein aggregates were detected using ProteoStat Aggresome Detection Kit (ENZ-51305-K100, Enzo). Samples were stained for 1 h at RT after secondary antibody incubation and washed profusely with PBS.

### Flow cytometry

ARPE-19 cells were washed with sterile PBS, trypsinized for 2–3 min in a humidified incubator at 37ºC, 5% CO_2_ and pelleted by centrifugation at 1200 × g for 5 min. Cells were resuspended in complete medium containing 5 μM DHE (D11347, Invitrogen), 5 μM DCF (D399, Invitrogen), 10 nM MitoTracker Deep Red (M22426, Invitrogen), 25 nM TMRM (T668, Invitrogen), 5 μM CellROX Deep Red (C10422, Invitrogen) or 5 μM MitoSOX Red (M36008, Invitrogen) and incubated for 15 min at 37ºC. DAPI (1 μg/μL) was added for viability assessment and tubes were kept in ice until analysis. Samples were analyzed using a CytoFlex S (V4-B2-Y4-R3; Beckman Coulter) flow cytometer and at least 10,000 events per sample were collected.

### RT-qPCR

Total RNA was isolated using RNeasy Mini Kit (74,106, QIAGEN) and retrotranscribed using High-Capacity RT Superscript Kit (4,374,966, Applied). Gene expression was determined using the following TaqMan Gene Expression assays (Thermo Fisher): *PINK1* (Hs00260868_m1)*, PARK2* (Hs01038318_m1), *BNIP3* (Hs00969291_m1), *BNIP3L* (Hs00188949_m1), *LAMP1* (Hs00931467_g1), *CTSA* (Hs00264902_m1), *CTSD* (Hs00157205_m1), *SQSTM1* (Hs00177654_m1), *UBE2QL1* (Hs00331876_m1), *ATG5* (Hs00355494_m1). Ribosomal *18S* (Hs99999901_s1) was used as reference gene and experiments were run using a LightCycler 480 platform (Roche).

### Selective autophagy assessment using tandem fluorescent reporters (MAP, *mito*-QC, tfGal3)

Samples were fixed using 3.7% PFA (50–980-487, EMS) containing 175 mM HEPES (15,630,056, Gibco) at pH 7.0 for 15 min (cells) or 2 h (whole eyes) at RT. In the case of cryosections, samples were air-dried for 5 min and rehydrated with PBS pH 7.0 before incubation with 1 µg/mL DAPI in PBS pH 7.0 for 15 min. Slides were washed 3 times with PBS pH 7.0 and mounted using VECTASHIELD (cryosections; H-1000–10, Vector Laboratories) or ProLong Diamond. Images were captured with a 0.5-µm z-step using a Leica TCS SP8 confocal microscope equipped with a 63 × immersion objective.

### Acidic lysosome staining and Cathepsin B activity assay

To assess lysosomal function, 1X MagicRed Cathepsin B fluorogenic substrate (938, ImmunoChemistry Technologies) or 1 µM LysoTracker Red DND-99 (L7528, Invitrogen) were added for the last 30 min of the treatment and cells were fixed for 10 min using 4% PFA. Nuclei were counterstained with 1 µg/mL DAPI for 15 min and/or processed for immunostaining and coverslips were mounted using ProLong Diamond. Images were captured with a 1-µm z-step using a Leica TCS SP8 confocal microscope equipped with a 63 × immersion objective.

### Image analysis

All image processing and analyses were performed using Fiji (ImageJ) and automatized whenever possible to avoid bias.

#### Retinal cell type quantification

The number of TUNEL^+^ cells, Brn3a^+^ RGCs, PKC^+^ bipolar interneurons, ConeArrestin^+^ cone photoreceptors, Iba1^+^ microglia, and GFAP^+^ astrocytic projections were manually quantified using *Cell Counter*. VisualArrestin^+^ OS length was manually measured and internalization was reported as the ratio between OS and IS + ONL mean fluorescence intensity in maximal projections.

#### Tandem fluorescence reporter analysis

*mito*-QC retinal cryosection and RPE flat mount images were pre-processed by subtracting background with a rolling ball radius of 25 pixels and applying a Gaussian blur filter with a Σ radius of 1. The same threshold was applied to each image to obtain GFP^+^ and mCherry^+^ masks. Next, the GFP^+^ mask was subtracted from the mCherry^+^ mask to isolate the signal corresponding to mitolysosomes. Finally, individual mitolysosomes were quantified using 3D Objects Counter [14]. Area of the GFP^+^ mask is reported as mitochondrial mass. In the case of ARPE-19 cell lines, *mito*-QC Counter or *auto*-QC Counter (MAP, tfGal3) were used as previously described [15].

#### Protein aggregate, autophagosome, p62 and 4-Hydroxynonenal accumulation

Images were pre-processed by subtracting background with a rolling ball radius of 15 pixels and applying a Gaussian blur filter with a Σ radius of 0.75. The same threshold was applied to each image to obtain 4-HNE^+^, ProteoStat^+^, p62^+^or LC3^+^ masks. Autophagosomes, p62, protein and 4-HNE^+^ aggregates were quantified using 3D Objects Counter [14]. In the case of cells and retinal cryosections, mean fluorescence intensity (MFI) of 4-HNE of the maximal projection is reported. A similar approach was used to quantify LAMP-2^+^ lysosomes in ARPE-19 cells.

#### Lysosomal parameters in ARPE-19 cells

The number of LysoTracker^+^, Galectin-3^+^ or MagicRed^+^ cells was quantified in at least 10 different images per coverslip and is reported as the percentage of positive cells per experiment (~ 150 cells in total).

### Statistical analysis

Sample size for mouse experiments was determined based on previous experimental designs from the lab. Data were evaluated for normality and heteroscedasticity. Normally distributed data were analyzed using a one-way or two-way ANOVA with post-hoc comparisons (more than two groups) or two-tailed Student’s *t*-test (two groups). Non-normally distributed data were analyzed using Kruskal–Wallis (more than two groups) or Mann–Whitney’s *U*-test (two groups). All statistical tests were performed with GraphPad Prism 9.0 and data were presented as the mean ± standard error of the mean (s.e.m).

## Results

### Urolithin A alleviates sodium iodate-induced neurodegeneration

Sodium iodate (SI) is an oxidizing agent whose administration mimics the late stages of dry AMD, including RPE cell death, geographic atrophy and loss of visual function [16]. Previous evidence in the literature suggests that SI might inhibit macroautophagy [17] concomitant with mitochondrial dysfunction [18]. With this in mind, we tested whether the well-described mitophagy inducer Urolithin A (UA) [19] could have neuroprotective benefits in the SI model. We first tested our hypothesis in an ex vivo organotypic eyecup culture system (Supplementary Fig. 1A), and observed that UA decreased the amount of F-actin cortex gaps between neighboring cells and preserved cytoskeleton integrity determined by F-actin thickness (Supplementary Fig. 1B), both phenotypes present in AMD patients [20]. The RPE provides trophic support to photoreceptors within the neuroretina, and is essential to ensure their survival [21]. Supporting our findings, we also detected that UA reduced 4-hydroxynonenal (4-HNE; lipid peroxidation byproduct) levels (Supplementary Fig. 1C) and apoptotic cell death in the photoreceptor-containing outer nuclear layer (ONL) of SI-treated retinas evaluated using TUNEL assay (Supplementary Fig. 1D).

**Fig. 1:**
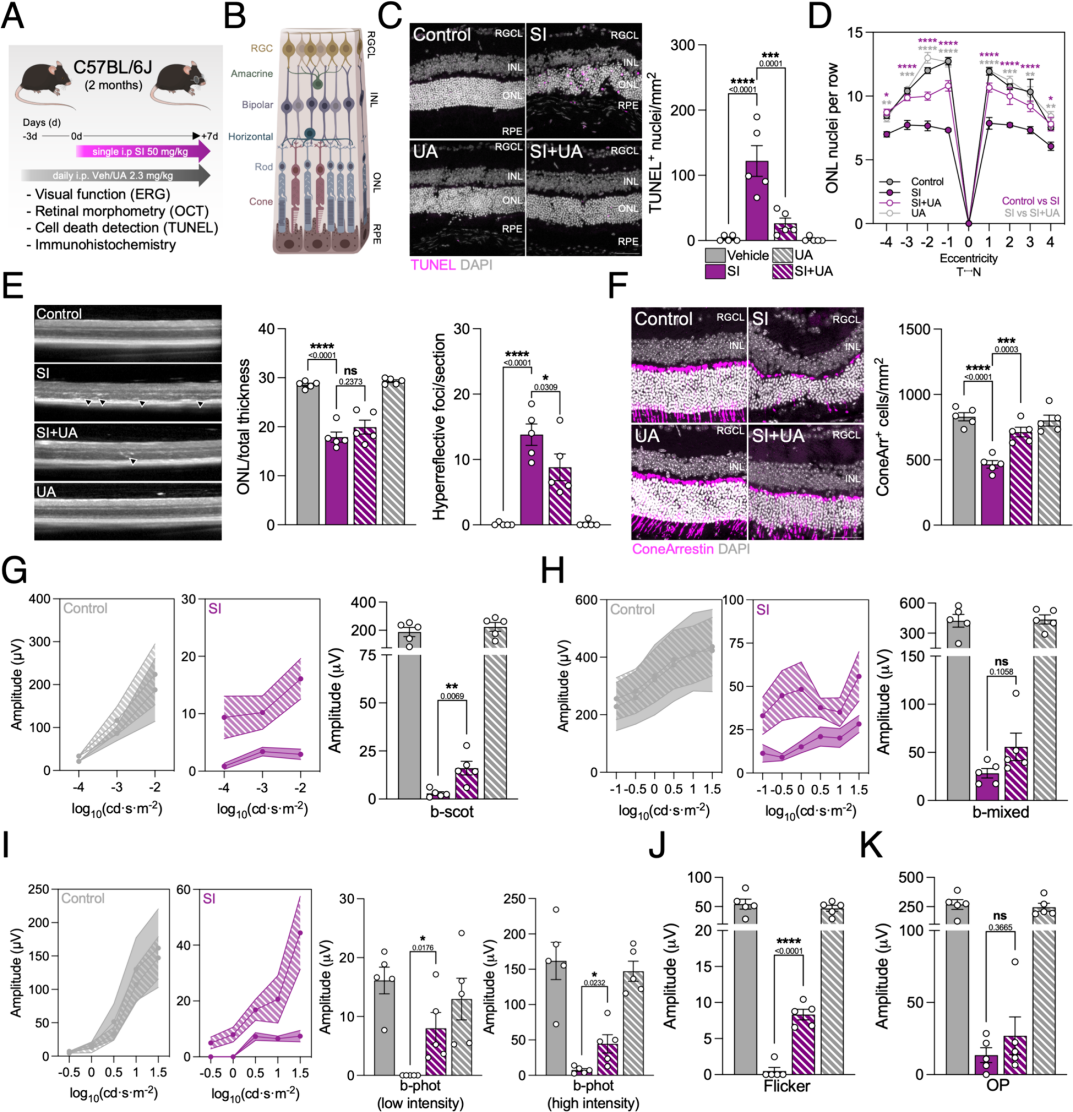
Urolithin A (UA) prevents photoreceptor cell death and preserves visual function in the sodium iodate (SI) model of geographic atrophy. **A** Young C57BL/6J mice (2 months) were administered UA (2.3/mg/kg/day) or Vehicle (DMSO; Veh, Control) intraperitoneally (i.p.) daily at day -3. At day 0, animals received a single i.p. injection of SI (50 mg/kg) and samples were collected on day 7. **B** Diagram depicting the different layers and cell types of the murine retina, including the retinal ganglion cell layer (RGCL), inner nuclear layer (INL) and outer nuclear layer (ONL). **C** Representative images and quantification of TUNEL assay (magenta) to detect apoptotic cell death in whole eye cryosections. Nuclei were counterstained with DAPI (gray). **D** Spider plot showing the number of nuclei per row in the photoreceptor-containing ONL. Control-SI and SI–SI+UA statistics are shown in magenta and gray, respectively. **E** Representative optical coherence tomography (OCT) and quantification of ONL thickness normalized to total retina (left) and AMD-associated hyperreflective foci (right). **F** Representative images and quantification of ConeArrestin^+^ cone photoreceptors (magenta), nuclei were counterstained with DAPI (gray). **G** Electroretinogram (ERG) assessment of visual function in scotopic (dark) conditions and quantification of b-wave amplitude (μV) at 0.01 cd·s·m^−2^. **H** ERG assessment of visual function in mixed conditions and quantification of b-wave amplitude (μV) at 31.62 cd·s·m^−2^. **I** ERG assessment of visual function in photopic (light) and quantification of b-wave amplitude (μV) at 1 cd·s·m^−2^ (low) and 31.62 cd·s·m^−2^ (high). **J** ERG assessment of photopic flicker response amplitude (μV) at 31.62 cd·s·m^−2^. **K** ERG assessment of oscillatory potential (OP) amplitude (μV) at 31.62 cd·s·m^−2^. Scale bars, 50 μm. All data are expressed as the mean ± s.e.m. Dots represent individual mice. *P* values were calculated using 1-way ANOVA with Šidak’s (**C**, **E**, **F**, **G**, **H**, **I**, **J**, **K**) or Tukey’s (**D**) post-hoc test. ****P < 0.0001, ***P < 0.001, **P < 0.01, *P < 0.05, ns: not significant

We next translated our experimental design to an in vivo setting using young C57BL/6J mice (2 months) that were treated i.p. daily with UA (2.3 mg/kg/day) for three days before SI (50 mg/kg) retinal degeneration induction. Animals continued to receive daily UA and did not present any overt side-effects up until they were sacrificed seven days later (Fig. 1A). Again, UA reduced SI-induced apoptotic cell death that was mainly restricted to the ONL and RPE (Fig. 1B, C). Morphometric analysis of the whole neuroretina (Fig. 1D) and optical coherence tomography (OCT; Fig. 1E, left) revealed increased thickness of the ONL after UA treatment supporting increased photoreceptor survival. UA also reduced the number of hyperreflective foci in the neuroretina and subretinal space (Fig. 1E, right), a phenomenon that has been linked to AMD progression [22]. Finally, we also observed an increased number of surviving cone photoreceptors in mice that received UA (Fig. 1F) as well as improved rod photoreceptor outer segment integrity (Supplementary Fig. 2A). No changes were observed in RGC number (Supplementary Fig. 2B) or bipolar interneuron number (Supplementary Fig. 2C, left), even though SI increased the number of protruded post-synaptic terminals (Supplementary Fig. 2C, right) which has been associated with synaptic function loss [22]. However, UA attenuated neuroinflammation as evidenced by decreased astrocytic gliosis (Supplementary Fig. 2D) and microglia infiltration and activation (Supplementary Fig. 2E).

Most importantly, daily UA administration alleviated vision loss in rod-driven scotopic (dark) conditions (Fig. 1G), mixed (Fig. 1H) and cone-driven photopic (light) conditions (Fig. 1I) measured using in vivo electroretinogram (ERG) evaluation. Flicker photopic response to repeated light stimuli was also preserved in UA treated mice (Fig. 1J), while no differences were observed in amacrine-driven ERG oscillatory potentials (OP, Fig. 1K). Even though the SI model induces very aggressive retinal degeneration [16], UA treatment helped maintain RPE integrity, bypassed photoreceptor cell death and, ultimately, preserved visual function. These findings suggest that UA might be eliciting the activation of pathways involved in the early response to SI-induced damage in the RPE, which we further explored using a combination of in vitro and in vivo approaches.

### Macroautophagy and proteostasis defects induced by SI are resolved after UA co-treatment

**Fig. 2:**
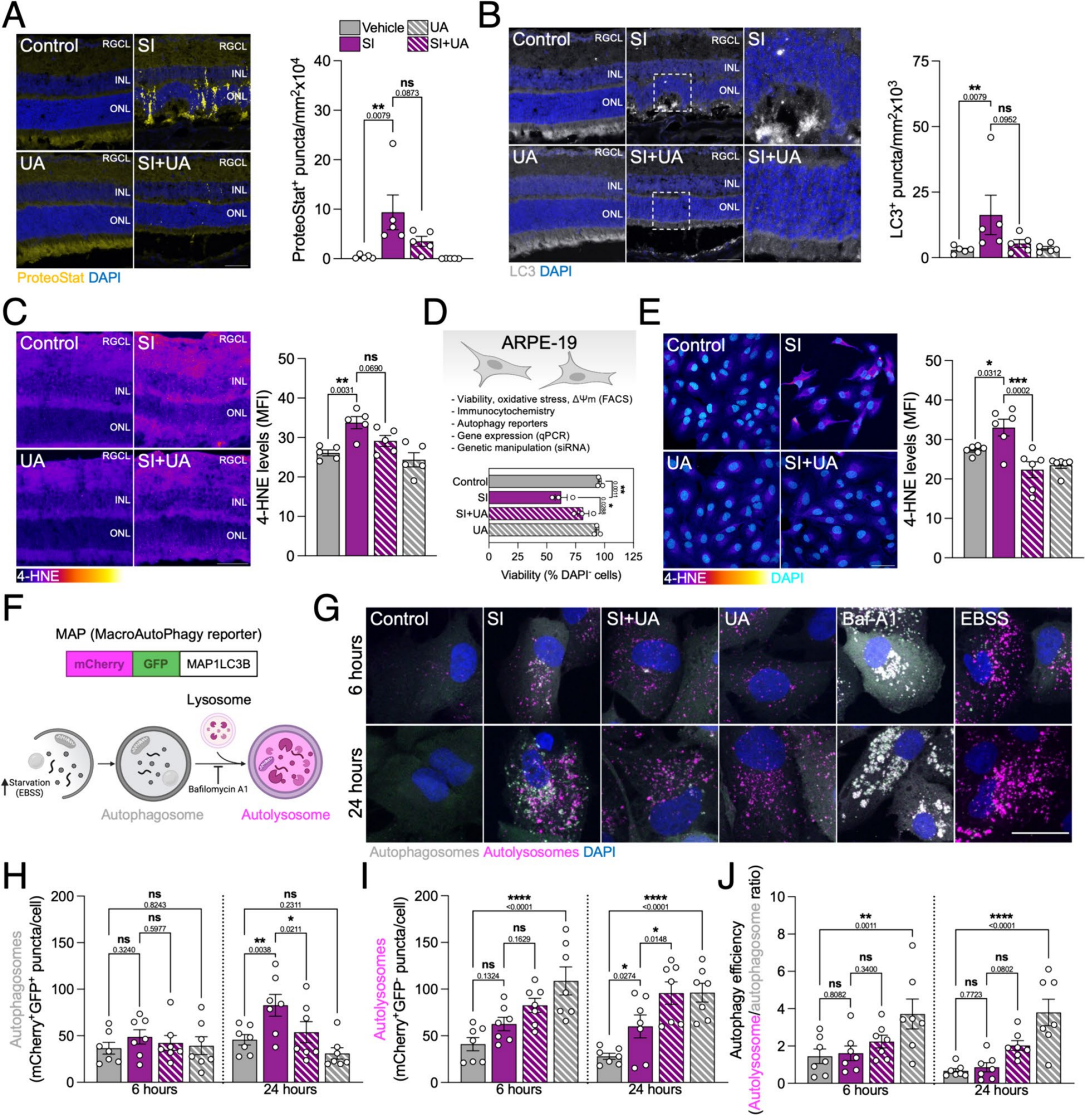
SI induces proteostasis defects in mice and ARPE-19 cells that are reversed by UA administration. **A** Representative images and quantification of ProteoStat assay (yellow) to detect protein aggregates in whole eye cryosections. Nuclei were counterstained with DAPI (blue). **B** Representative images and quantification of LC3^+^ autophagosomes (gray) in whole eye cryosections, nuclei were counterstained with DAPI (blue). **C** Representative images and quantification of 4-hydroxynonenal (4-HNE) levels (Fire LUT) in whole eye cryosections. **D** Human-derived ARPE-19 cells were treated with 20 mM SI and/or 100 µM UA for 24 h. Viability was evaluated by nuclear exclusion assay (DAPI) using flow cytometry. **E** Representative images and quantification of 4-hydroxynonenal (4-HNE) levels (Fire LUT) in ARPE-19 cells. **F** Diagram depicting the basis of MAP macroautophagy tandem fluorescent reporter assay. **G** Representative images of ARPE-19 cells expressing the MAP reporter, 100 nM Bafilomycin A1 (Baf-A1) and EBSS were used as negative and positive controls, respectively. Autophagosomes (mCherry^+^GFP^+^, gray) and autolysosomes (mCherry^+^GFP^−^, magenta) can be observed, nuclei were counterstained with DAPI (blue). **H** Quantification of the number of autophagosomes as shown in **G**. **I** Quantification of the number of autolysosomes as shown in **G**. **J** Autophagy efficiency is reported as the number of autolysosomes per autophagosome, indicative of autophagic flux. Scale bars, 50 μm (**A**, **B**,** C**,** E**) and 25 μm (**G**). All data are expressed as the mean ± s.e.m. Dots represent individual mice (**A**, **B**,** C**), independent experiments (**E**, **D**) or biological replicates from three independent experiments (**H**, **I**, **J**). *P* values were calculated using Kruskal–Wallis with Dunn’s (**A**, **B**), 1-way ANOVA with Šídák’s (**C**, **E**), Tukey’s (**D**) or Fisher’s LSD (**H**, **I**, **J**) *post-hoc* test. ****P < 0.0001, ***P < 0.001, **P < 0.01, *P < 0.05, ns: not significant

Following up on previous evidence [17], ProteoStat assay revealed a very severe accumulation of protein aggregates in the retina after SI treatment (Fig. 2A), that was concomitant with an accumulation of LC3^+^ autophagosomes (Fig. 2B) and lipid peroxidation-derived 4-HNE adducts (Fig. 2C); all of these observations were partially or fully reverted by UA co-treatment. In order to further dissect the molecular mechanisms implicated in SI-triggered proteostasis defects and neuroprotection with UA, we used human-derived ARPE-19 cells as an in vitro model (Fig. 2D, top). Replicating our findings ex vivo and in vivo, UA also restored viability (Fig. 2D, bottom) and reduced 4-HNE levels in cells subjected to SI (Fig. 2E). Interestingly, UA did not reduce cytosolic O_2_^·^, H_2_O_2_ or OH^·^ levels (Supplementary Fig. 3A-C) pointing to the compound exerting its effect downstream of ROS production.

**Fig. 3:**
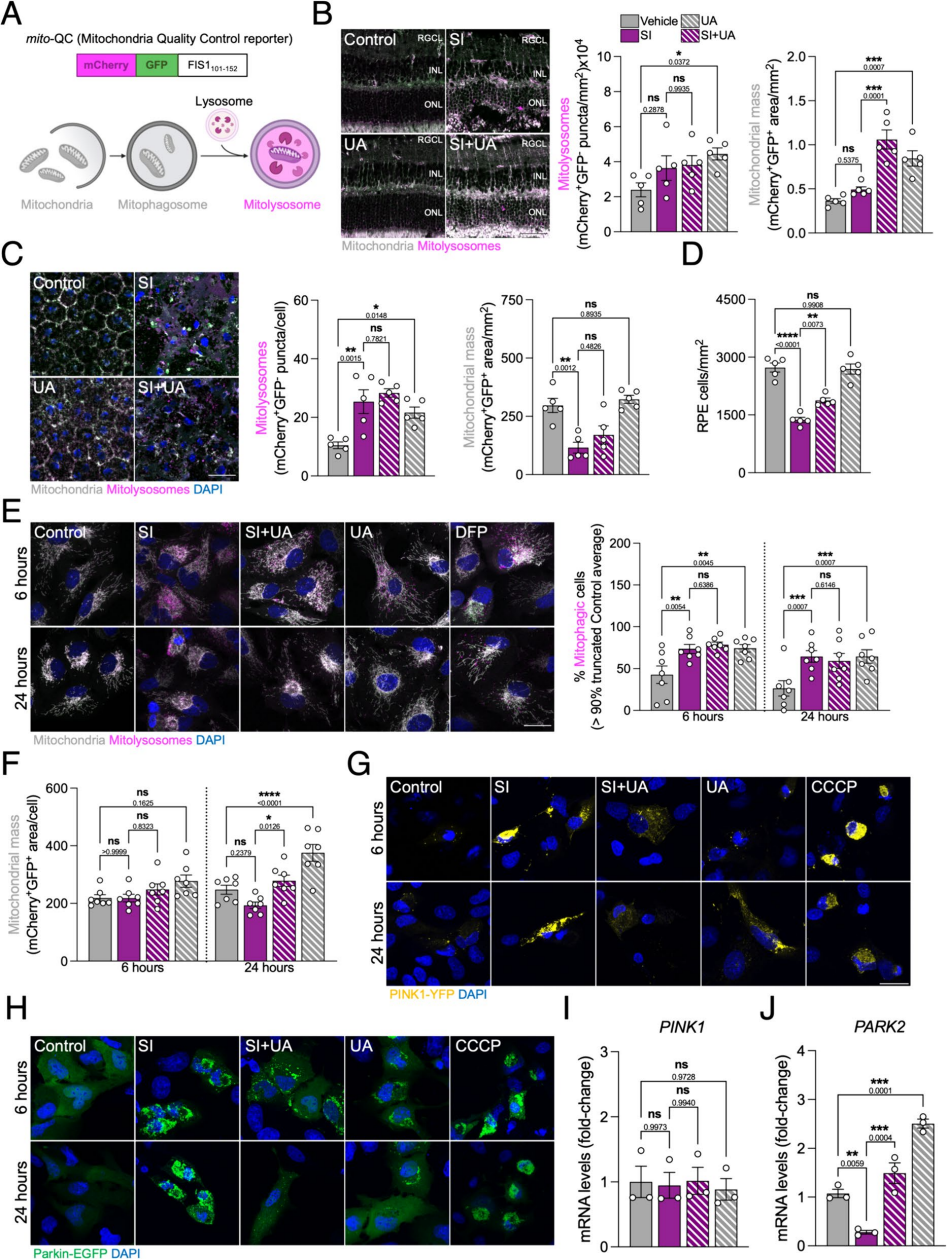
Both SI and UA stimulate PINK1/Parkin-dependent mitophagy. **A** Diagram depicting the basis of *mito*-QC mitophagy tandem fluorescent reporter assay. **B** Representative images and quantification of whole eye cryosections of C57BL/6J mice expressing the *mito*-QC reporter and treated with SI and/or UA as previously described. Mitochondria (mCherry^+^GFP^+^, gray) and mitolysosomes (mCherry^+^GFP^−^, magenta) can be observed, nuclei were counterstained with DAPI (blue). **C** Representative images and quantification of mitophagy and mitochondrial mass in RPE flat mounts. **D** Subplasmalemmal mitochondria localization allows for RPE cell counting, as shown in **C**. **E** Representative images and quantification of mitophagy in ARPE-19 cells expressing the *mito*-QC reporter, 1 mM deferiprone (DFP) was used as a positive control. **G** Quantification of mitochondrial mass in ARPE-19 cells as shown in **C**. **G** Representative images of ARPE-19 cells expressing PINK1-YFP (yellow), nuclei were counterstained with DAPI (blue). **H** Representative images of ARPE-19 cells expressing Parkin-EGFP (green), nuclei were counterstained with DAPI (blue). **I-J** Expression levels of *PINK1* (**I**) and *PARK2* (**J**) mRNA in ARPE-19 cells, evaluated by RT-qPCR. Scale bars, 50 μm (**B**,** C**) and 25 μm (**E**, **G**, **H**). All data are expressed as the mean ± s.e.m. Dots represent individual mice (**B**, **C**, **D**) (**A**, **B**, **C**, **D**), biological replicates from three independent experiments (**E**, **F**) (**H**, **I**, **J**) or independent experiments (**I**, **J**). *P* values were calculated using 1-way ANOVA with Šídák’s (**B**, **C**, **D**, **I**, **J**) or Fisher’s LSD (**E**, **F**) post-hoc test. ****P < 0.0001, ***P < 0.001, **P < 0.01, *P < 0.05, ns: not significant

Autophagy is a very dynamic process that requires precise tools for its monitoring [21]. Taking advantage of the different pH sensitivity of fluorescent proteins, we evaluated non-selective macroautophagy using the MAP reporter that consists of a chimeric protein including pH-insensitive mCherry, pH-sensitive GFP and MAP1LC3B [23] (Fig. 3F). Therefore, autophagosomes can be identified as mCherry^+^GFP^+^ (gray) vesicles while autolysosomes will be mCherry^+^GFP^−^ (magenta). Both SI and UA stimulated autophagy (Fig. 2G), but in the case of SI there was an increase in the number of autophagosomes (Fig. 2H) concomitant to increased autolysosome count (Fig. 2I), indicative of defective autophagy flux. Treatment with UA restored autophagosome delivery to the lysosome defects caused by SI, as evidenced by an increased autolysosome/autophagosome ratio (Fig. 2J). These data point to UA being able to maintain proteostasis via autophagy restoration in SI-challenged animals and cells.

### UA restores mitochondrial homeostasis in SI-challenged cells by stimulating mitophagy and mitochondrial biogenesis

Since UA had initially been described as a robust mitophagy inducer [19], we evaluated its effect in vivo using the tandem fluorescent reporter *mito*-QC [23] (Fig. 3A). Following a similar principle, *mito*-QC is a chimeric protein including mCherry, GFP and the mitochondria-targeting sequence of FIS1 (FIS1_101-152_) that allows us to differentiate between mCherry^+^GFP^+^ cytoplasmic mitochondria (gray) and mCherry^+^GFP^−^ mitolysosomes (magenta). Indeed, UA induced mitophagy in the neuroretina (Fig. 3B) and RPE (Fig. 3C). SI significantly stimulated mitophagy in the RPE (Fig. 3C), but no robust changes were observed in the neuroretina (Fig. 3B). It has been previously reported that UA also has the ability to induce mitochondrial biogenesis [24] but, while we observed an increase in mitochondrial mass in the neuroretina (Fig. 3B), no changes were observed in the RPE (Fig. 3C). Decisively, subplasmalemmal localization of mitochondria within the RPE allows for individual cell counting and we validated that UA increased RPE survival in SI-treated mice (Fig. 3D).

A similar phenotype was observed in ARPE-19 cells, whereby UA induced simultaneously mitophagy (Fig. 3E) and mitochondrial biogenesis (Fig. 3F, Supplementary Fig. 4A). SI treatment resulted in mitophagy stimulation as early as 6 h (Fig. 3E) supporting the hypothesis of mitophagy being a protective mechanism at earlier stages of neurodegeneration. Indeed, SI induced mitochondrial superoxide production (Supplementary Fig. 4B) and mitochondrial membrane hyperpolarization (Supplementary Fig. 4C) and these were slightly reduced when cells were co-treated with UA suggesting that mitochondrial homeostasis is restored.

Previous reports have linked UA to activation of the PINK1/Parkin-dependent mitophagy pathway [25]. ARPE-19 cells endogenously express detectable levels of Parkin and, accordingly, UA triggered moderate PINK1-YFP (Fig. 3G) and Parkin-EGFP condensation (Fig. 3H). SI induced severe PINK1 and Parkin accumulation that increased over time but was resolved by UA addition (Fig. 3G, H). These findings suggest that SI might also be inducing mitophagy flux blockage. No changes were observed regarding *PINK1* mRNA levels (Fig. 3I) but *PARK2*/Parkin expression was downregulated in response to SI and upregulated by UA (Fig. 3J). Since *PARK2* is also under transcriptional regulation of other stress-responsive pathways, namely ATF4 [26], these changes might be unrelated to mitophagy. No expressions changes were observed in either *BNIP3* or *BNIP3L*/NIX (Supplementary Fig. 4D), key effectors in receptor-mediated mitophagy.

### Lysosomal membrane permeabilization is a hallmark of SI-induced cell death

**Fig. 4:**
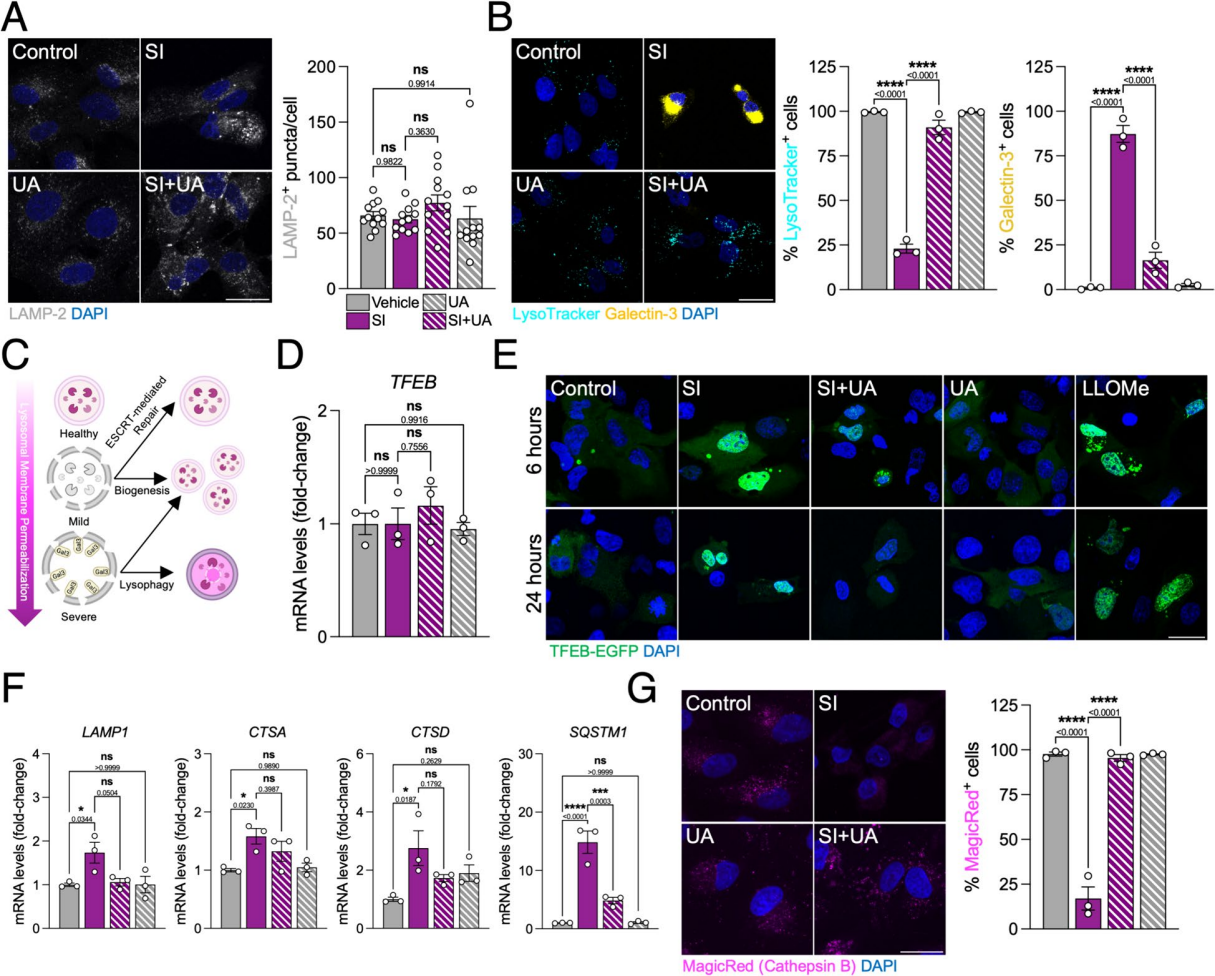
SI induces severe lysosomal membrane permeabilization (LMP) and activation of the CLEAR network. **A** Representative images and quantification of immunostained LAMP-2^+^ lysosomes (gray) in ARPE-19 cells treated for 24 h, nuclei were counterstained with DAPI (blue). **B** Representative images and quantification of the number of cells presenting acidic (LysoTracker^+^, cyan) and/or permeabilized (Galectin-3^+^, yellow) lysosomes, nuclei were counterstained with DAPI (blue). **C** Diagram depicting the distinct cellular responses to lysosomal damage and LMP. **D** Representative images of ARPE-19 cells expressing TFEB-EGFP (green) to assess nuclear translocation, nuclei were counterstained with DAPI (blue). **F** Gene expression levels of genes under transcriptional control of TFEB (CLEAR network; *LAMP1, CTSA, CTSD, SQSTM1*) in ARPE-19 cells, evaluated by RT-qPCR**. G** Representative images and quantification of the number of cells with Cathepsin B activity (MagicRed, magenta), nuclei were counterstained with DAPI (blue). Scale bars, 25 μm. All data are expressed as the mean ± s.e.m. Dots represent biological replicates from three independent experiments (**A**) or independent experiments (**B**, **D, F, G**). *P* values were calculated using 1-way ANOVA with Šídák’s *post-hoc* test. ****P < 0.0001, ***P < 0.001, *P < 0.05, ns: not significant

Altogether, these data pointed to autophagic cargo being accumulated and not degraded in response to SI. Lysosomal alterations have previously been observed in AMD patients [6], so we investigated whether this was also the case in the SI model. While we observed no differences on lysosome number, they appeared to be enlarged in response to SI (Fig. 4A). However, SI induced a decrease in the number of acidic lysosomes that was overcome by UA treatment (Fig. 4B; cyan). One of the main causes for loss of acidic pH is lysosomal membrane permeabilization (LMP), which can be evaluated by assessing cytosolic Galectin-3 recruitment to the luminal lysosome glycocalyx [27]. Indeed, SI induced severe LMP that was fully rescued by UA (Fig. 4B; yellow).

Cells have different mechanisms to deal with LMP depending on damage severity (Fig. 4C), including membrane repair, lysosomal biogenesis or, ultimately, recycling via lysophagy [28]. We did not detect any changes on mRNA levels of the transcription factor TFEB, which acts as the master regulator of lysosomal biogenesis (Fig. 4D). However, using EGFP-tagged TFEB we observed that SI triggered its translocation to the nucleus (Fig. 4E) and the subsequent upregulation of downstream genes (*LAMP1, CTSA, CTSB, SQSTM1*; Fig. 4F), collectively known as the CLEAR network [28]. No changes were observed in cells that were treated simultaneously with UA (Fig. 4E, F) but lysosomal function, evaluated using a fluorogenic Cathepsin B substrate, was fully restored (Fig. 4G). These data suggest that UA aids to overcome defective lysosome function caused by SI independently of TFEB-dependent lysosomal biogenesis.

### p62-dependent lysophagy is essential for the pro-survival effect of UA

**Fig. 5:**
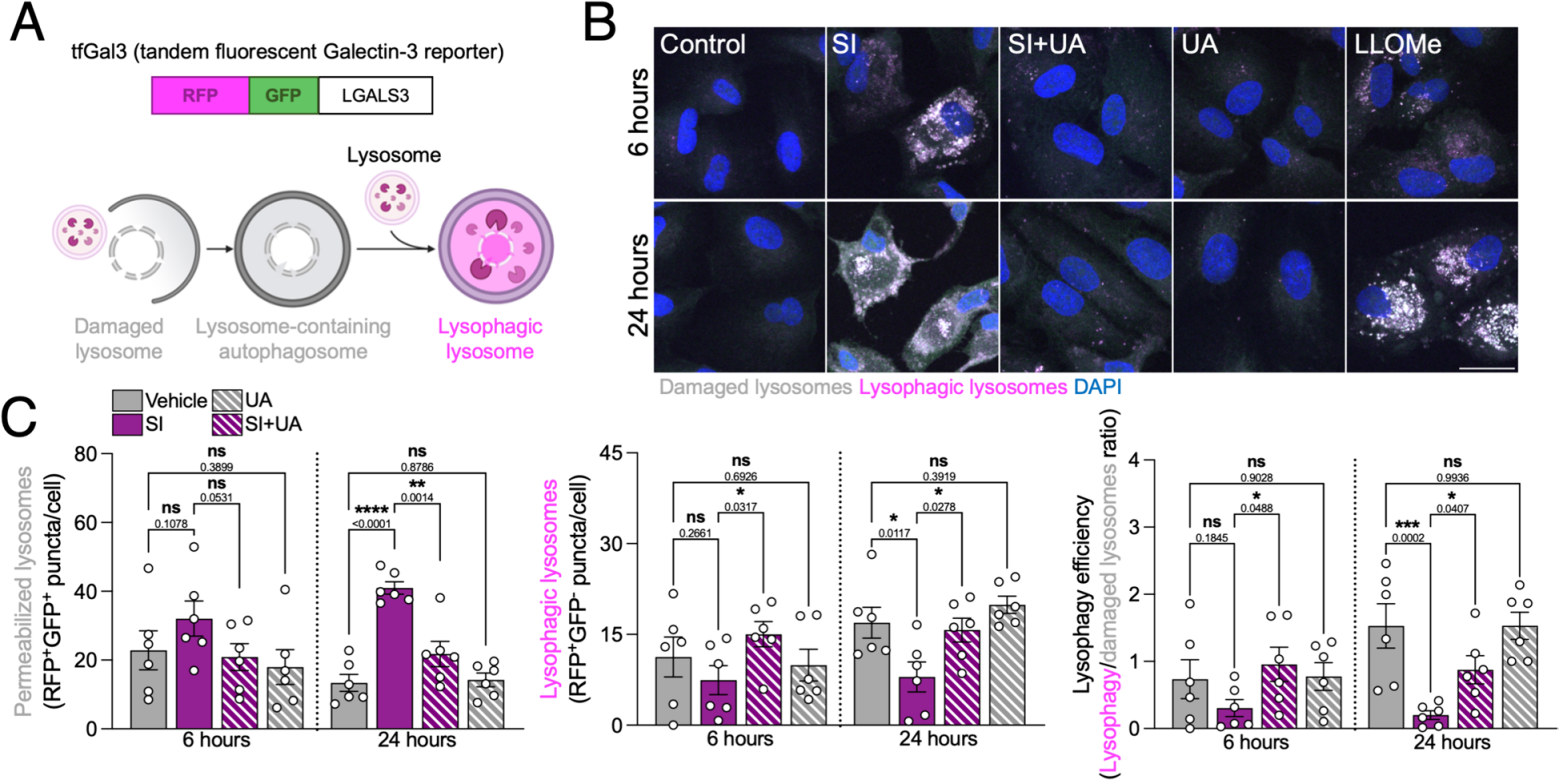
UA restores upcycling of damaged lysosomes via lysophagy. **A** Diagram depicting the basis of tfGal3 lysophagy tandem fluorescent reporter assay. **B** Representative images of ARPE-19 cells expressing the tfGal3 reporter, 1 mM LLOMe was used as positive control. Permeabilized lysosomes (RFP^+^GFP^+^, gray) and lysophagic lysosomes (RFP^+^GFP^−^, magenta) can be observed, nuclei were counterstained with DAPI (blue). **C** Quantification of the number of damaged lysosomes and lysophagic lysosomes as shown in **B**. Lysophagy efficiency is reported as the number of lysophagy events per permeabilized lysosome, indicative of lysophagic flux. Scale bar, 25 μm. All data are expressed as the mean ± s.e.m. Dots represent biological replicates from three independent experiments. *P* values were calculated using 1-way ANOVA with Fisher’s LSD *post-hoc* test. ****P < 0.0001, ***P < 0.001, **P < 0.01, *P < 0.05, ns: not significant

Given the high levels of LMP, and the fact that lysosomal biogenesis was not stimulated by UA, we evaluated upcycling of lysosomes via lysophagy. We assessed lysophagy in ARPE-19 cells using the tandem fluorescent reporter tfGal3 [29] (Fig. 5A), a construct composed of pH-insensitive RFP, pH-sensitive GFP and LGALS3, that allows us to differentiate between RFP^+^GFP^+^ permeabilized lysosomes (gray) and RFP^+^GFP^−^ lysophagic events (magenta) (Fig. 5B**)**. We validated that SI also triggered exogenous Galectin-3 accumulation that was fully reverted by UA addition (Fig. 5C, left). Interestingly, SI induced a time-dependent decrease in the number of lysophagic lysosomes that was abrogated by UA (Fig. 5C, middle and right).

**Fig. 6:**
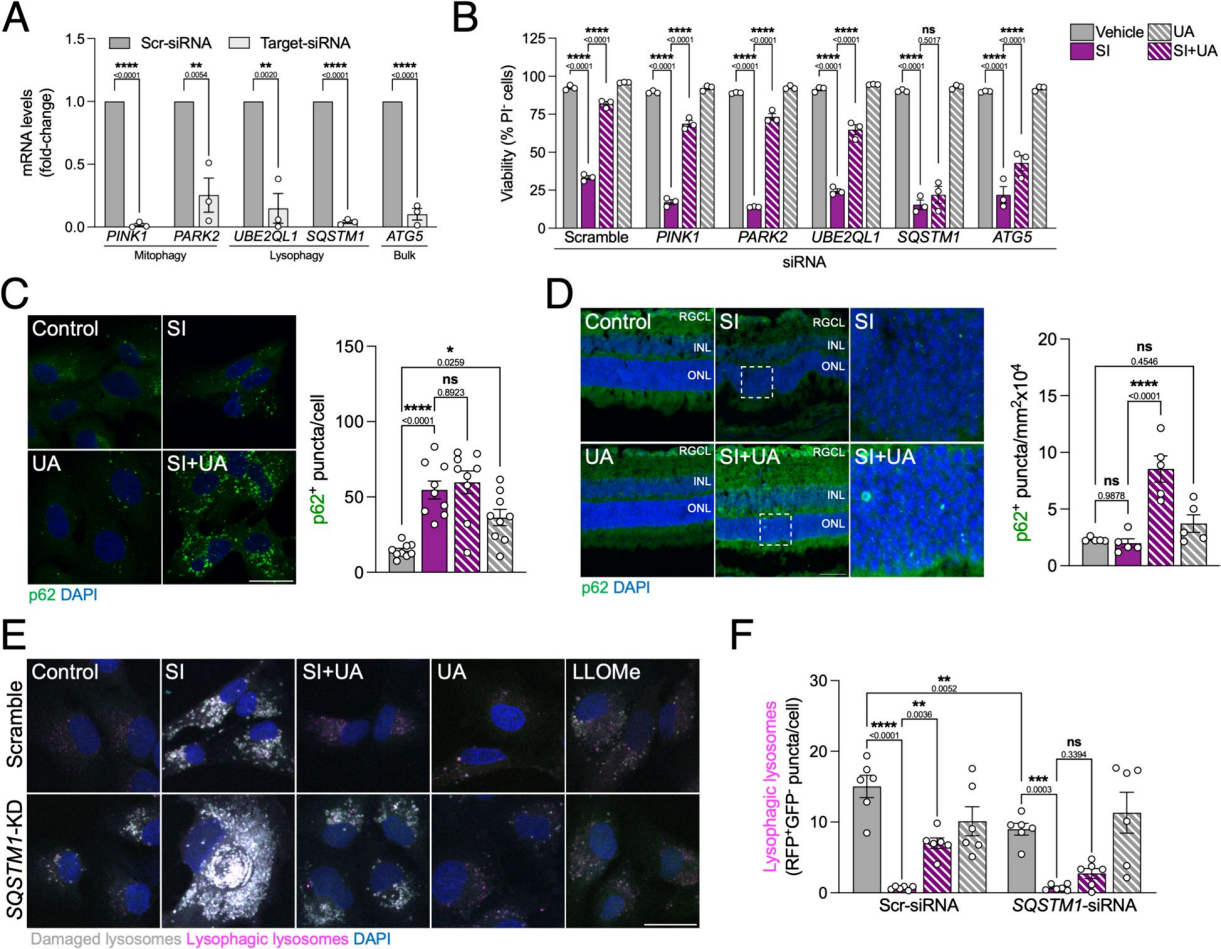
p62-dependent lysophagy is essential for the neuroprotective effect of UA. **A** Knockdown of mitophagy (*PINK1*, *PARK2*), lysophagy (*UBE2QL1*, *SQSTM1*) or bulk autophagy (*ATG5*) in ARPE-19 cells was achieved through targeted siRNA lipofection. Knockdown efficiency was validated using RT-qPCR. **B** Viability assessment by nuclear exclusion assay (propidium iodide, PI) in ARPE-19 cells treated for 24 h using flow cytometry, selective autophagy regulators were genetically downregulated as shown in **A**. **C** Representative images and quantification of immunostained p62^+^ puncta (green) in ARPE-19 cells, nuclei were counterstained with DAPI (blue). **D** Representative images and quantification of immunostained p62^+^ puncta (green) in whole eye cryosections, nuclei were counterstained with DAPI (blue). **E** Representative images of ARPE-19 cells expressing the tfGal3 reporter and transfected with either Scramble- or *SQSTM1*-targeting siRNA (*SQSTM1*-KD), 1 mM LLOMe was used as positive control. Permeabilized lysosomes (RFP^+^GFP^+^, gray) and lysophagic lysosomes (RFP^+^GFP^−^, magenta) can be observed, nuclei were counterstained with DAPI (blue). **F** Quantification of lysophagic lysosomes as shown in **E**. Scale bars, 25 μm. All data are expressed as the mean ± s.e.m. Dots represent independent experiments (**A**, **B**) or biological replicates from three independent experiments (**C**, **D**, **F**). *P* values were calculated using unpaired Student’s *t*-test (**A**), 1-way ANOVA with Šídák’s (**C**, **D**) or Fisher’s LSD (**F**) *post-hoc* test. ****P < 0.0001, ***P < 0.001, **P < 0.01, *P < 0.05, ns: not significant

Selective autophagy pathways such as mitophagy have been extensively studied in the literature, but evidence on lysophagy regulators is scarcer. Recently, conflicting reports have described the ubiquitin-conjugating enzyme UBE2QL1 [30] and the ubiquitin adaptor SQSTM1/p62 [31] as essential for lysophagy execution. Since UA simultaneously induced macroautophagy (Fig. 2G, I), mitophagy (Fig. 3D) and lysophagy (Fig. 5B,C), we genetically knocked down (KD) their key effectors (*PINK1*, *PARK2* for mitophagy; *UBE2QL1*, *SQSTM1* for lysophagy; *ATG5* for bulk macroautophagy) to determine which is the main contributor to UA-mediated neuroprotection (Fig. 6A). Surprisingly, only *SQSTM1*-KD fully abrogated viability rescue by in SI-treated ARPE-19 cells (Fig. 6B). p62 has been described to form condensates in damaged lysosomal membranes, acting as a signaling platform to recruit autophagy machinery and initiate autophagosome formation leading to lysophagy [31]. As previously shown, SI induced a 15-fold increase in *SQSTM1* mRNA levels (Fig. 4F) and we similarly observed an increase at the protein level (Fig. 6C). In vivo, p62 increase was only observed in SI-treated mice that received UA (Fig. 6D), reinforcing the notion that p62 might play a key role in alleviating early cellular stress and preventing cell death in this model. Using the *mito*-QC reporter, *SQSTM1*-KD had no effect on mitophagy induction by UA, thus discarding it as the main driver of its pro-survival role (Supplementary Fig. 5A,B,F). Macroautophagy reporter MAP cells revealed that UA was still able to partially restore unspecific autophagic flux (Supplementary Fig. 5C-F). Combination of *SQSTM1*-KD and the lysophagy reporter tfGal3 revealed that p62 downregulation decreased basal lysophagy, exacerbated permeabilized lysosome accumulation and fully abrogated restoration of lysophagic flux by UA in cells treated with SI (Fig. 6E,F, Supplementary Fig. 5F).

Taken together, our findings highlight LMP as a key player in SI-induced geographic atrophy and retinal neurodegeneration. Stimulation of damaged lysosome upcycling via lysophagy using UA promotes RPE survival and alleviates visual function loss caused by SI.

## Discussion

Lipofuscin is an autofluorescent pigment that progressively accumulates inside lysosomes throughout physiological aging, reducing their degradation efficiency [32]. Age-associated lipofuscin accumulation is more prominent in the RPE, as it has to additionally deal with debris and byproducts from recycling photoreceptor outer segments through the visual cycle [10]. Exacerbated lipofuscin deposition has been observed in AMD patients [33] as well as in the SI model [34]. Recently, it was described that lipofuscin buildup can crystallize and damage the lysosomal membrane, leading to LMP, in a mouse model of impaired visual pigment processing (*Abca4*^*−/−*^*Rdh8*^*−/−*^) [35]. Previous research has also shown that iron-loaded ferritin accumulates inside the lysosomes of SI-treated ARPE-19 cells, leading to impaired hydrolase (Cathepsin D) maturation [17]. Our data highlights that SI similarly triggers severe LMP and upregulates the CLEAR network transcriptional program to stimulate lysosomal biogenesis, but is not enough to sustain cell homeostasis and ensure RPE survival. We observed a robust increase in hydrogen peroxide levels in response to SI, that could freely diffuse into the lysosomes [36] and catalyze Fenton-like reactions inside their iron-rich lumen, eventually leading to lysosomal membrane damage and LMP. Whether iron or lipofuscin accumulation is responsible for LMP and deficient autophagy in AMD or SI-induced geographic atrophy still remains to be elucidated.

Recent efforts have focused on deciphering how lysophagy, the degradation of damaged lysosomes within healthy lysosomes, is initiated and carried out. A targeted siRNA screen of human E2 ubiquitin-conjugating enzymes found UBE2QL1 to be essential for damaged lysosome K48-linked ubiquitination and recruitment of the autophagy adaptors p62 and TAX1BP1 [30]. Using proximity biotinylation proteomics, another group found that only TAX1BP1, and its upstream kinase TBK1, was required to increase lysophagic flux in response to lysosomal damage [37]. An independent study using the tandem fluorescent reporter TMEM192-mKeima validated the role of TBK1 signaling in lysophagy and revealed that the E2 ubiquitin-conjugating enzymes UBE2L3 and UBE2N are also involved. However, while there was a decrease in Galectin-3 puncta, the authors did not find a downregulation of lysophagy in *SQSTM1*-KD cells [38]. In contrast, another group found that only p62 was essential and specifically required for lysophagy, forming phase condensates surrounding lysosomal membrane damage site. Notably, previously non-understood ALS-associated mutations in p62 (L341V, P392L, G425R) impaired lysophagy [31]. In our experimental setting, UBE2QL1 was dispensable for UA-dependent viability rescue in ARPE-19 cells treated with SI but *SQSTM1*-KD fully abrogated it via impairing lysophagy. Further research is required to address these apparently conflicting results, extent of LMP or cell type-specific mechanisms could elicit different responses or signaling pathways that converge in lysophagy.

Supporting our findings, evidence from the literature shows that low-dose SI treatment (500 μM) in ARPE-19 cells induces lysosomal enlargement and increases the levels of p62 [34]. Another study observed that autophagy inhibition using either 3-methyladenine (3-MA; PI3K inhibitor) or Bafilomycin A1 (lysosomal acidification, autolysosome fusion inhibitor) further sensitizes ARPE-19 cells to SI-induced cell death [18]. Rapamycin, a traditional autophagy inducer, has accordingly shown neuroprotective benefits against SI treatment in vitro [34] and in vivo [39]. Even though current research highlights promising results regarding lifespan extension and therapeutic potential, the use of rapamycin in the clinic and its putative side effects are still being debated [40].

UA is a natural polyphenolic small molecule derived from ellagitannins, found in pomegranate, nuts and berries. This compound was initially postulated as a potent mitophagy inducer with the ability to extend lifespan and healthspan in animal models [19], showing also positive outcomes and improved mitochondrial homeostasis in its first clinical trials in middle-aged [41] and elderly [24] participants. Recent studies have focused on the impact of UA in neurodegenerative diseases. Treatment with UA induces mitophagy in microglia to bolster phagocytosis in amyloid-ß and tau pathology, decreasing cognitive decline in Alzheimer’s disease animal models [42]. In a similar model, UA was found to stimulate neurogenesis as well as reducing neuronal cell death [43]. We validated previously published evidence [19, 25] as UA also induced autophagy and PINK1/Parkin-dependent mitophagy in the neuroretina, RPE and ARPE-19 cells. Surprisingly, we found that UA is also able to restore lysosomal function after LMP by promoting p62-dependent lysophagy. These findings open up a plethora of new therapeutic applications for UA. Indeed, it was recently shown that administration of ellagic acid, its precursor, reduced lysosomal alterations in models of Niemann-Pick disease, a rare inherited disease that is characterized by aberrant lipid handling within lysosomes [44].

Regarding clinical dry AMD management, there are currently no *gold standard* treatments. In the past decade, two different complement inhibitors that can be delivered intravitreally have received regulatory approval (*Pegcetacoplan*, *Avacincaptad pegol*) and shown moderate but positive outcomes in slowing down the progression of geographic atrophy [45, 46]. Their efficacy in a larger cohort of patients, and whether it is influenced by the prevalent high-risk CFH^Y402H^ polymorphism [47], remains to be elucidated. Moreover, while it is routinely used in the clinic, recurrent intravitreal injections can increase the risk of suffering other ocular pathologies [48]. In a recent study, we demonstrated that UA is able to reach the central nervous system after intraperitoneal administration [49] and it has also shown beneficial neuroprotective effects after oral administration in other models of neurodegeneration [42]. Therefore, the use of UA could overrule the need for intravitreal administration of currently available treatments. We also show that, besides autophagy, UA also alleviates other RPE cellular hallmarks of AMD such as lysosomal alterations [6], altered mitochondrial dynamics [50] and function [51], or secondary retinal neuroinflammation [49, 52].

Due to its favorable pharmacokinetic profile and bioavailability, together with being a readily available natural compound, UA warrants further exploration as a neuroprotective agent in AMD and other ocular diseases. Moreover, induction of lysophagy, with UA or yet-to-be-discovered novel drugs, represents a new therapeutic window for diseases involving lysosomal dysfunction such as lysosomal storage disorders (LSDs), sarcopenia or non-alcoholic fatty liver disease (NAFLD) [53].

## Conclusions

In summary, SI induces LMP in vitro and in vivo causing autophagy flux blockage evidenced by autophagosome, lipid peroxidation adducts and protein aggregate accumulation. Treatment with UA induced PINK1/Parkin-dependent mitophagy and improved mitochondrial homeostasis in SI-treated cells, but this process is dispensable for its pro-survival effect in our experimental context. Surprisingly, UA decreased LMP, restored lysosomal function and autophagy flux by promoting lysophagy. siRNA-mediated knockdown experiments revealed that UA rescues viability in cells treated with SI via p62-dependent lysophagy, Ultimately, UA prevented SI-induced geographic atrophy and RPE cell death, preserving photoreceptor homeostasis and visual function in vivo.

### Supplementary Information

Supplementary Material 1.

## Data Availability

No datasets were generated within the present manuscript. Source data are available from the corresponding authors upon reasonable request.

## Abbreviations

4-HNE: 4-Hydroxynonenal
ALS: Amyotrophic lateral sclerosis
AMD: Age-related macular degeneration
ATF4: Activating transcription factor 4
CLEAR: Coordinated lysosomal expression and regulation
DAPI: 4′,6-Diamidino-2-phenylindole
DHE: Dihydroethdium
DCF: 2',7'-Dichlorofluorescein
DMEM: Dulbecco’s Modified Eagle’s Medium
ERG: Electroretinography
GFP: Green fluorescent protein
HEPES: 4-(2-Hydroxyethyl)-1-piperazineethanesulfonic acid
LAMP-1: Lysosome-associated membrane protein 1
LAMP-2: Lysosome-associated membrane protein 2
LC3: Microtubule-associated proteins 1A/1B light chain 3B
LMP: Lysosomal membrane permeabilization
NGS: Normal goat serum
OCT: Optical coherence tomography
ONL: Outer nuclear layer
OP: Oscillatory potentials
PBS: Phosphate-buffered saline
PFA: Paraformaldehyde
PINK1: PTEN-induced kinase 1
RFP: Red fluorescent protein
RPE: Retinal pigment epithelium
SI: Sodium iodate, NaIO_3_
SQSTM1/p62: Sequestosome-1
TAX1BP1: Tax1-binding protein 1
TBK1: TANK-binding kinase 1
TFEB: Transcription factor EB
TMRM: Tetramethylrhodamine, methyl ester
TUNEL: Terminal deoxynucleotidyl transferase dUTP nick end labeling
UA: Urolithin A
UBE2QL1: Ubiquitin Conjugating Enzyme E2 Q Family Like 1
VEGF: Vascular endothelial growth factor

## References

[1] Vyawahare H, Shinde P (2022). Age-related macular degeneration: epidemiology, pathophysiology, diagnosis, and treatment. Cureus.

[2] Wong WL, Su X, Li X, Cheung CM, Klein R, Cheng CY, Wong TY (2014). Global prevalence of age-related macular degeneration and disease burden projection for 2020 and 2040: a systematic review and meta-analysis. Lancet Glob Health.

[3] Villarejo-Zori B, Jimenez-Loygorri JI, Zapata-Munoz J, Bell K, Boya P (2021). New insights into the role of autophagy in retinal and eye diseases. Mol Aspects Med.

[4] Vargas JNS, Hamasaki M, Kawabata T, Youle RJ, Yoshimori T (2023). The mechanisms and roles of selective autophagy in mammals. Nat Rev Mol Cell Biol..

[5] Ye F, Kaneko H, Hayashi Y, Takayama K, Hwang SJ, Nishizawa Y, Kimoto R, Nagasaka Y, Tsunekawa T, Matsuura T (2016). Malondialdehyde induces autophagy dysfunction and VEGF secretion in the retinal pigment epithelium in age-related macular degeneration. Free Radic Biol Med.

[6] Golestaneh N, Chu Y, Xiao YY, Stoleru GL, Theos AC (2017). Dysfunctional autophagy in RPE, a contributing factor in age-related macular degeneration. Cell Death Dis.

[7] Ferrington DA, Ebeling MC, Kapphahn RJ, Terluk MR, Fisher CR, Polanco JR, Roehrich H, Leary MM, Geng Z, Dutton JR, Montezuma SR (2017). Altered bioenergetics and enhanced resistance to oxidative stress in human retinal pigment epithelial cells from donors with age-related macular degeneration. Redox Biol.

[8] Lakkaraju A, Umapathy A, Tan LX, Daniele L, Philp NJ, Boesze-Battaglia K, Williams DS (2020). The cell biology of the retinal pigment epithelium. Prog Retin Eye Res.

[9] Zhang Y, Cross SD, Stanton JB, Marmorstein AD, Le YZ, Marmorstein LY (2017). Early AMD-like defects in the RPE and retinal degeneration in aged mice with RPE-specific deletion of Atg5 or Atg7. Mol Vis.

[10] Kim JY, Zhao H, Martinez J, Doggett TA, Kolesnikov AV, Tang PH, Ablonczy Z, Chan CC, Zhou Z, Green DR, Ferguson TA (2013). Noncanonical autophagy promotes the visual cycle. Cell.

[11] Ramírez-Pardo I, Villarejo-Zori B, Jiménez-Loygorri JI, Sierra-Filardi E, Alonso-Gil S, Mariño G, de la Villa P, Fitze PS, Fuentes JM, García-Escudero R (2023). Ambra1 haploinsufficiency in CD1 mice results in metabolic alterations and exacerbates age-associated retinal degeneration. Autophagy..

[12] Notomi S, Ishihara K, Efstathiou NE, Lee JJ, Hisatomi T, Tachibana T, Konstantinou EK, Ueta T, Murakami Y, Maidana DE (2019). Genetic LAMP2 deficiency accelerates the age-associated formation of basal laminar deposits in the retina. Proc Natl Acad Sci USA.

[13] McWilliams TG, Prescott AR, Allen GF, Tamjar J, Munson MJ, Thomson C, Muqit MM, Ganley IG (2016). mito-QC illuminates mitophagy and mitochondrial architecture in vivo. J Cell Biol.

[14] Bolte S, Cordelieres FP (2006). A guided tour into subcellular colocalization analysis in light microscopy. J Microsc.

[15] Montava-Garriga L, Singh F, Ball G, Ganley IG (2020). Semi-automated quantitation of mitophagy in cells and tissues. Mech Ageing Dev.

[16] Chowers G, Cohen M, Marks-Ohana D, Stika S, Eijzenberg A, Banin E, Obolensky A (2017). Course of sodium iodate-induced retinal degeneration in albino and pigmented mice. Invest Ophthalmol Vis Sci.

[17] Ashok A, Chaudhary S, Wise AS, Rana NA, McDonald D, Kritikos AE, Lindner E, Singh N (2021). Release of iron-loaded ferritin in sodium iodate-induced model of age related macular degeneration: an in-vitro and in-vivo study. Antioxidants (Basel).

[18] Chan CM, Huang DY, Sekar P, Hsu SH, Lin WW (2019). Reactive oxygen species-dependent mitochondrial dynamics and autophagy confer protective effects in retinal pigment epithelial cells against sodium iodate-induced cell death. J Biomed Sci.

[19] Ryu D, Mouchiroud L, Andreux PA, Katsyuba E, Moullan N, Nicolet-Dit-Felix AA, Williams EG, Jha P, Lo Sasso G, Huzard D (2016). Urolithin A induces mitophagy and prolongs lifespan in C. elegans and increases muscle function in rodents. Nat Med.

[20] Tarau IS, Berlin A, Curcio CA, Ach T (2019). The cytoskeleton of the retinal pigment epithelium: from normal aging to age-related macular degeneration. Int J Mol Sci.

[21] Jiménez-Loygorri JI, Benítez-Fernández R, Viedma-Poyatos Á, Zapata-Muñoz J, Villarejo-Zori B, Gómez-Sintes R, Boya P (2023). Mitophagy in the retina: viewing mitochondrial homeostasis through a new lens. Prog Retin Eye Res.

[22] Duic C, Pfau K, Keenan TDL, Wiley H, Thavikulwat A, Chew EY, Cukras C (2023). Hyperreflective foci in age-related macular degeneration are associated with disease severity and functional impairment. Ophthalmol Retina.

[23] McWilliams TG, Prescott AR, Villarejo-Zori B, Ball G, Boya P, Ganley IG (2019). A comparative map of macroautophagy and mitophagy in the vertebrate eye. Autophagy.

[24] Andreux PA, Blanco-Bose W, Ryu D, Burdet F, Ibberson M, Aebischer P, Auwerx J, Singh A, Rinsch C (2019). The mitophagy activator urolithin A is safe and induces a molecular signature of improved mitochondrial and cellular health in humans. Nat Metab.

[25] Luan P, D'Amico D, Andreux PA, Laurila PP, Wohlwend M, Li H, Imamura de Lima T, Place N, Rinsch C, Zanou N, Auwerx J (2021). Urolithin A improves muscle function by inducing mitophagy in muscular dystrophy. Sci Transl Med.

[26] Bouman L, Schlierf A, Lutz AK, Shan J, Deinlein A, Kast J, Galehdar Z, Palmisano V, Patenge N, Berg D (2011). Parkin is transcriptionally regulated by ATF4: evidence for an interconnection between mitochondrial stress and ER stress. Cell Death Differ.

[27] Wang F, Gomez-Sintes R, Boya P (2018). Lysosomal membrane permeabilization and cell death. Traffic.

[28] Papadopoulos C, Kravic B, Meyer H (2020). Repair or lysophagy: dealing with damaged lysosomes. J Mol Biol.

[29] Maejima I, Takahashi A, Omori H, Kimura T, Takabatake Y, Saitoh T, Yamamoto A, Hamasaki M, Noda T, Isaka Y, Yoshimori T (2013). Autophagy sequesters damaged lysosomes to control lysosomal biogenesis and kidney injury. Embo J.

[30] Koerver L, Papadopoulos C, Liu B, Kravic B, Rota G, Brecht L, Veenendaal T, Polajnar M, Bluemke A, Ehrmann M (2019). The ubiquitin-conjugating enzyme UBE2QL1 coordinates lysophagy in response to endolysosomal damage. EMBO Rep.

[31] Gallagher ER, Holzbaur ELF (2023). The selective autophagy adaptor p62/SQSTM1 forms phase condensates regulated by HSP27 that facilitate the clearance of damaged lysosomes via lysophagy. Cell Rep.

[32] Kaarniranta K, Uusitalo H, Blasiak J, Felszeghy S, Kannan R, Kauppinen A, Salminen A, Sinha D, Ferrington D (2020). Mechanisms of mitochondrial dysfunction and their impact on age-related macular degeneration. Prog Retin Eye Res.

[33] Sparrow JR (2010). Bisretinoids of RPE lipofuscin: trigger for complement activation in age-related macular degeneration. Adv Exp Med Biol.

[34] Lin YC, Horng LY, Sung HC, Wu RT (2018). Sodium iodate disrupted the mitochondrial-lysosomal axis in cultured retinal pigment epithelial cells. J Ocul Pharmacol Ther.

[35] Pan C, Banerjee K, Lehmann GL, Almeida D, Hajjar KA, Benedicto I, Jiang Z, Radu RA, Thompson DH, Rodriguez-Boulan E, Nociari MM (2021). Lipofuscin causes atypical necroptosis through lysosomal membrane permeabilization. Proc Nat Acad Sci U S A.

[36] Brunk UT, Zhang H, Roberg K, Öllinger K (1995). Lethal hydrogen peroxide toxicity involves lysosomal iron-catalyzed reactions with membrane damage. Redox Rep.

[37] Eapen VV, Swarup S, Hoyer MJ, Paulo JA, Harper JW (2021). Quantitative proteomics reveals the selectivity of ubiquitin-binding autophagy receptors in the turnover of damaged lysosomes by lysophagy. Elife.

[38] Shima T, Ogura M, Matsuda R, Nakamura S, Jin N, Yoshimori T, Kuma A (2023). The TMEM192-mKeima probe specifically assays lysophagy and reveals its initial steps. J Cell Biol.

[39] Niu Z, Shi Y, Li J, Qiao S, Du S, Chen L, Tian H, Wei L, Cao H, Wang J, Gao L (2021). Protective effect of rapamycin in models of retinal degeneration. Exp Eye Res.

[40] Selvarani R, Mohammed S, Richardson A (2021). Effect of rapamycin on aging and age-related diseases-past and future. Geroscience.

[41] Singh A, D'Amico D, Andreux PA, Fouassier AM, Blanco-Bose W, Evans M, Aebischer P, Auwerx J, Rinsch C (2022). Urolithin A improves muscle strength, exercise performance, and biomarkers of mitochondrial health in a randomized trial in middle-aged adults. Cell Rep Med.

[42] Fang EF, Hou Y, Palikaras K, Adriaanse BA, Kerr JS, Yang B, Lautrup S, Hasan-Olive MM, Caponio D, Dan X (2019). Mitophagy inhibits amyloid-beta and tau pathology and reverses cognitive deficits in models of Alzheimer's disease. Nat Neurosci.

[43] Gong Z, Huang J, Xu B, Ou Z, Zhang L, Lin X, Ye X, Kong X, Long D, Sun X (2019). Urolithin A attenuates memory impairment and neuroinflammation in APP/PS1 mice. J Neuroinflammation.

[44] Soto-Huelin B, Babiy B, Pastor O, Díaz-García M, Toledano-Zaragoza A, Frutos MD, Espín JC, Tomás-Barberán FA, Busto R, Ledesma MD (2023). Ellagic acid and its metabolites urolithins A/B ameliorate most common disease phenotypes in cellular and mouse models for lysosomal storage disorders by enhancing extracellular vesicle secretion. Neurobiol Dis.

[45] Heier JS, Lad EM, Holz FG, Rosenfeld PJ, Guymer RH, Boyer D, Grossi F, Baumal CR, Korobelnik JF, Slakter JS (2023). Pegcetacoplan for the treatment of geographic atrophy secondary to age-related macular degeneration (OAKS and DERBY): two multicentre, randomised, double-masked, sham-controlled, phase 3 trials. Lancet.

[46] Khanani AM, Patel SS, Staurenghi G, Tadayoni R, Danzig CJ, Eichenbaum DA, Hsu J, Wykoff CC, Heier JS, Lally DR (2023). Efficacy and safety of avacincaptad pegol in patients with geographic atrophy (GATHER2): 12-month results from a randomised, double-masked, phase 3 trial. Lancet.

[47] Landowski M, Kelly U, Klingeborn M, Groelle M, Ding JD, Grigsby D, Bowes Rickman C (2019). Human complement factor H Y402H polymorphism causes an age-related macular degeneration phenotype and lipoprotein dysregulation in mice. Proc Natl Acad Sci USA.

[48] Falavarjani KG, Nguyen QD (2013). Adverse events and complications associated with intravitreal injection of anti-VEGF agents: a review of literature. Eye (Lond).

[49] Jiménez-Loygorri JI, Villarejo-Zori B, Viedma-Poyatos Á, Zapata-Muñoz J, Benítez-Fernández R, Frutos-Lisón MD, Tomás-Barberán FA, Espín JC, Area-Gómez E, Gomez-Duran A, Boya P (2024). Mitophagy curtails cytosolic mtDNA-dependent activation of cGAS/STING inflammation during aging. Nat Commun.

[50] Fisher CR, Shaaeli AA, Ebeling MC, Montezuma SR, Ferrington DA (2022). Investigating mitochondrial fission, fusion, and autophagy in retinal pigment epithelium from donors with age-related macular degeneration. Sci Rep.

[51] Ebeling MC, Polanco JR, Qu J, Tu C, Montezuma SR, Ferrington DA (2020). Improving retinal mitochondrial function as a treatment for age-related macular degeneration. Redox Biol.

[52] Kuchroo M, DiStasio M, Song E, Calapkulu E, Zhang L, Ige M, Sheth AH, Majdoubi A, Menon M, Tong A (2023). Single-cell analysis reveals inflammatory interactions driving macular degeneration. Nat Commun.

[53] Settembre C, Perera RM (2024). Lysosomes as coordinators of cellular catabolism, metabolic signalling and organ physiology. Nat Rev Mol Cell Biol..

