# Supplementary material for: Urolithin A promotes p62-dependent lysophagy to prevent acute retinal neurodegeneration
Source: Mol Neurodegener. 2024 Jun 18;19:49. doi: 10.1186/s13024-024-00739-3 (PMC11186080; doi:10.1186/s13024-024-00739-3)
Supplement: Supplementary file 1 — Supplementary Material 1. [file 13024_2024_739_MOESM1_ESM.docx]

**Urolithin A promotes p62-dependent lysophagy to prevent acute retinal neurodegeneration**

**Authors:** Juan Ignacio Jiménez-Loygorri^1*^, Álvaro Viedma-Poyatos^1^, Raquel Gómez-Sintes^1^, Patricia Boya^1,2*^

**Affiliations:**

^1^Department of Cellular and Molecular Biology, Centro de Investigaciones Biológicas Margarita Salas, CSIC, Madrid, Spain

^2^Department of Neuroscience and Movement Science, Faculty of Science and Medicine, University of Fribourg, Switzerland

**Corresponding author:* [*juan.ignacio.jimenez@cib.csic.es*](mailto:juan.ignacio.jimenez@cib.csic.es)*;* [*patricia.boya@unifr.ch*](mailto:patricia.boya@unifr.ch)

**Supplementary Materials** including:

- Supplementary Figures 1 to 5
- Supplementary Tables 1 and 2

**Supplementary Figures**

**
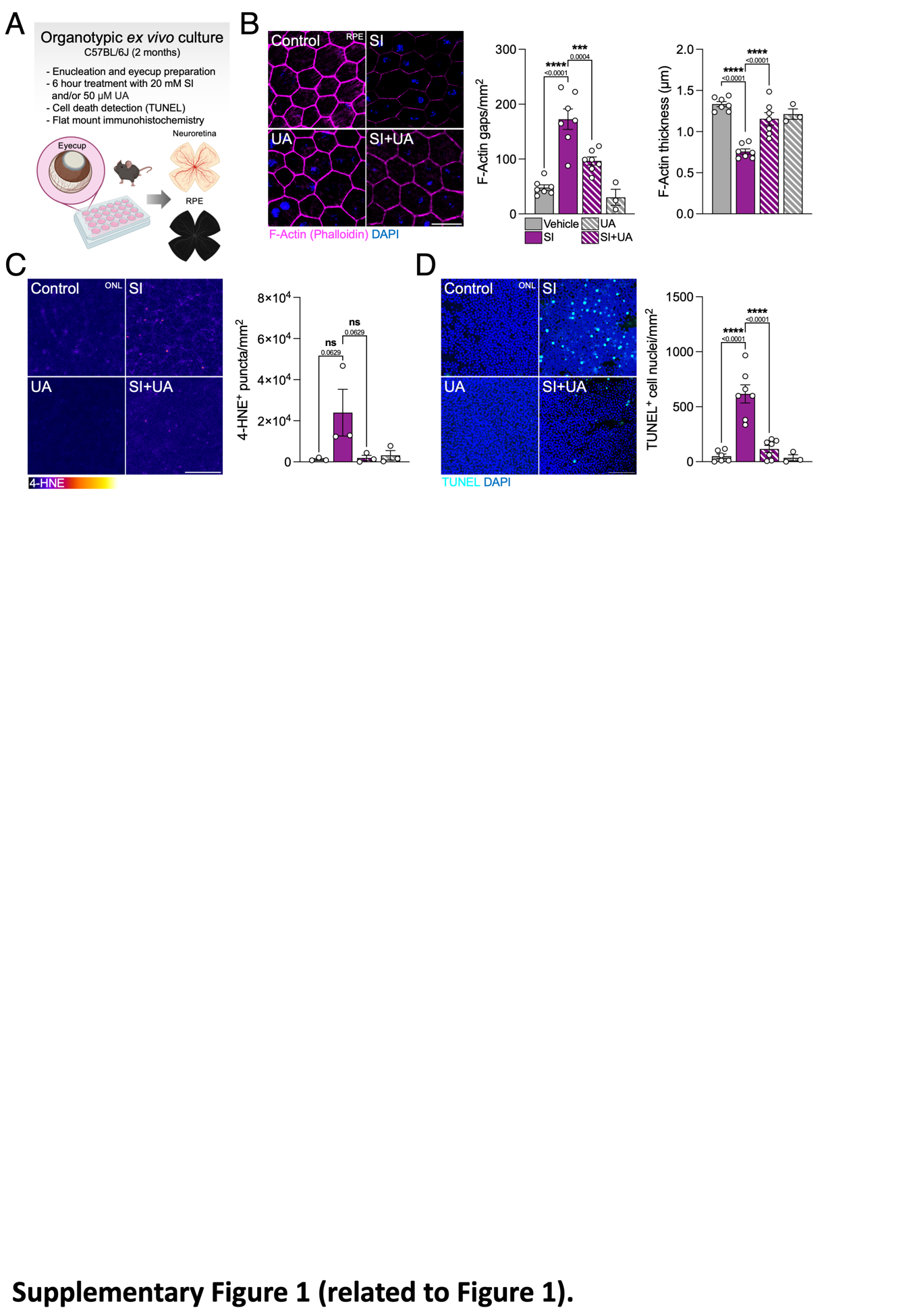
Supplementary Figure 1. UA shows neuroprotective effect in an *ex vivo* model of AMD. A** Eyes from young (2 months) C57BL/6J mice were enucleated, the cornea and lens removed, and cultured for 6 hours with 20 mM SI and/or 50 μM UA. **B** Representative images and quantification of F-Actin staining (Phalloidin; magenta) to assess cytoskeleton integrity in RPE flat mounts, nuclei were counterstained with DAPI (blue). **C** Representative images and quantification of 4-hydroxynonenal (4-HNE) levels (Fire LUT) in neuroretina flat mounts. **D** Representative images and quantification of TUNEL assay (cyan) to detect apoptotic cell death in neuroretina flat mounts, nuclei were counterstained with DAPI (blue). Scale bars, 25 μm (**B**), 50 μm (**C**) and 50 μm (**D**). All data are expressed as the mean ± s.e.m. Dots represent individual mice. *P* values were calculated using 1-way ANOVA with Šídák’s (**B**, **D**) or Kruskal-Wallis with Dunn’s (**C**) *post-hoc* test. *****P*<0.0001, ****P*<0.001, ns: not significant.

**
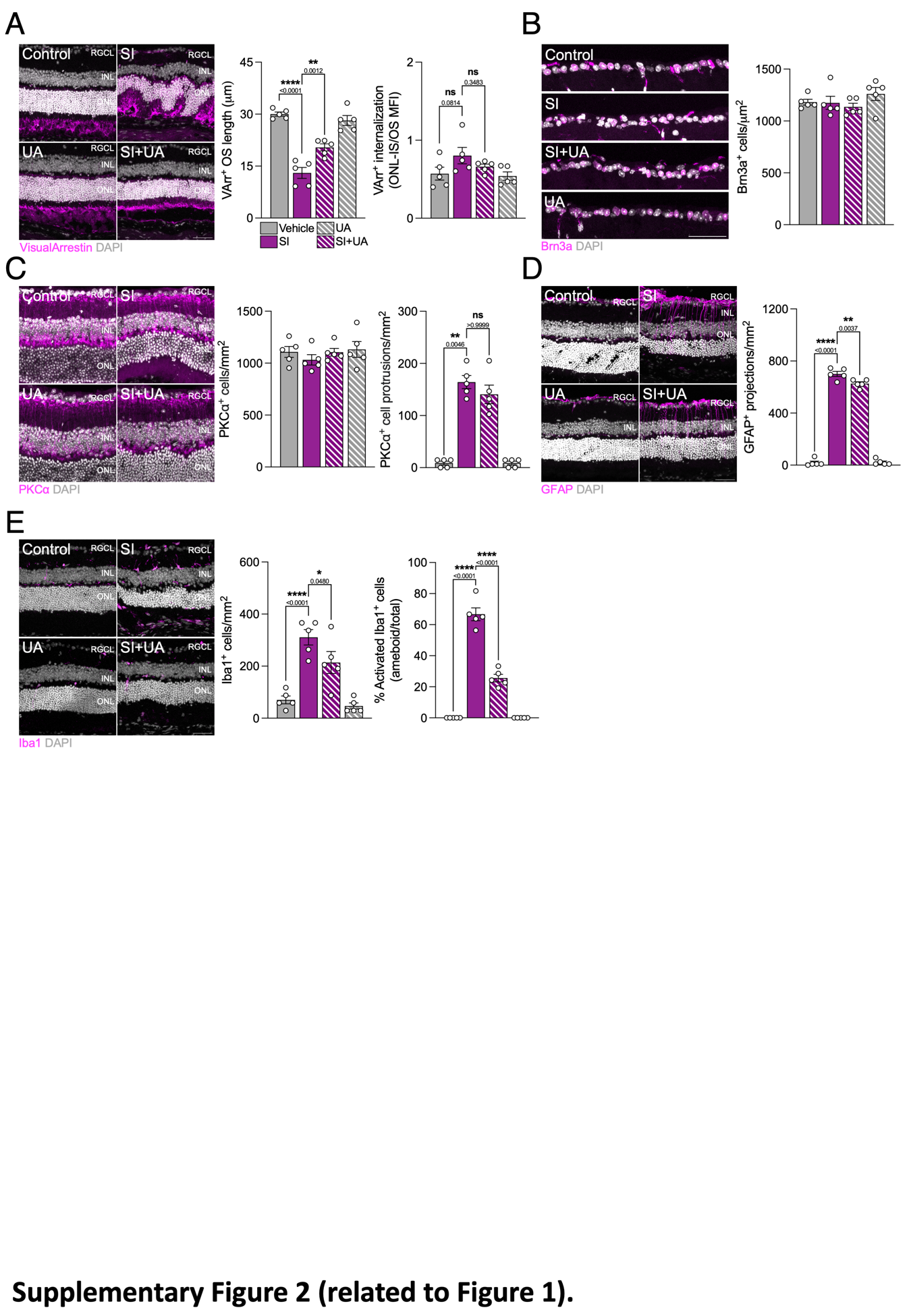
Supplementary Figure 2. UA preserves rod integrity and decreases SI-induced neuroinflammation. A** Representative images and quantification of VisualArrestin^+^ rod photoreceptors (magenta) length and internalization, nuclei were counterstained with DAPI (gray). **B** Representative images and quantification of Brn3a^+^ RGCs (magenta), nuclei were counterstained with DAPI (gray). **C** Representative images and quantification of PKCα^+^ bipolar interneurons (magenta), nuclei were counterstained with DAPI (gray). **D** Representative images and quantification of GFAP^+^ astrocytes (magenta), nuclei were counterstained with DAPI (gray). **E** Representative images and quantification of Iba1^+^ microglia (magenta) number and activation (ameboid/total), nuclei were counterstained with DAPI (gray). Scale bars, 50 μm. All data are expressed as the mean ± s.e.m. Dots represent individual mice. *P* values were calculated using 1-way ANOVA with Šídák’s (**A**, **B**, **C** left, **D**, **E**) or Kruskal-Wallis with Dunn’s (**C** right) *post-hoc* test. *****P*<0.0001, ***P*<0.01, **P*<0.05, ns: not significant.

**
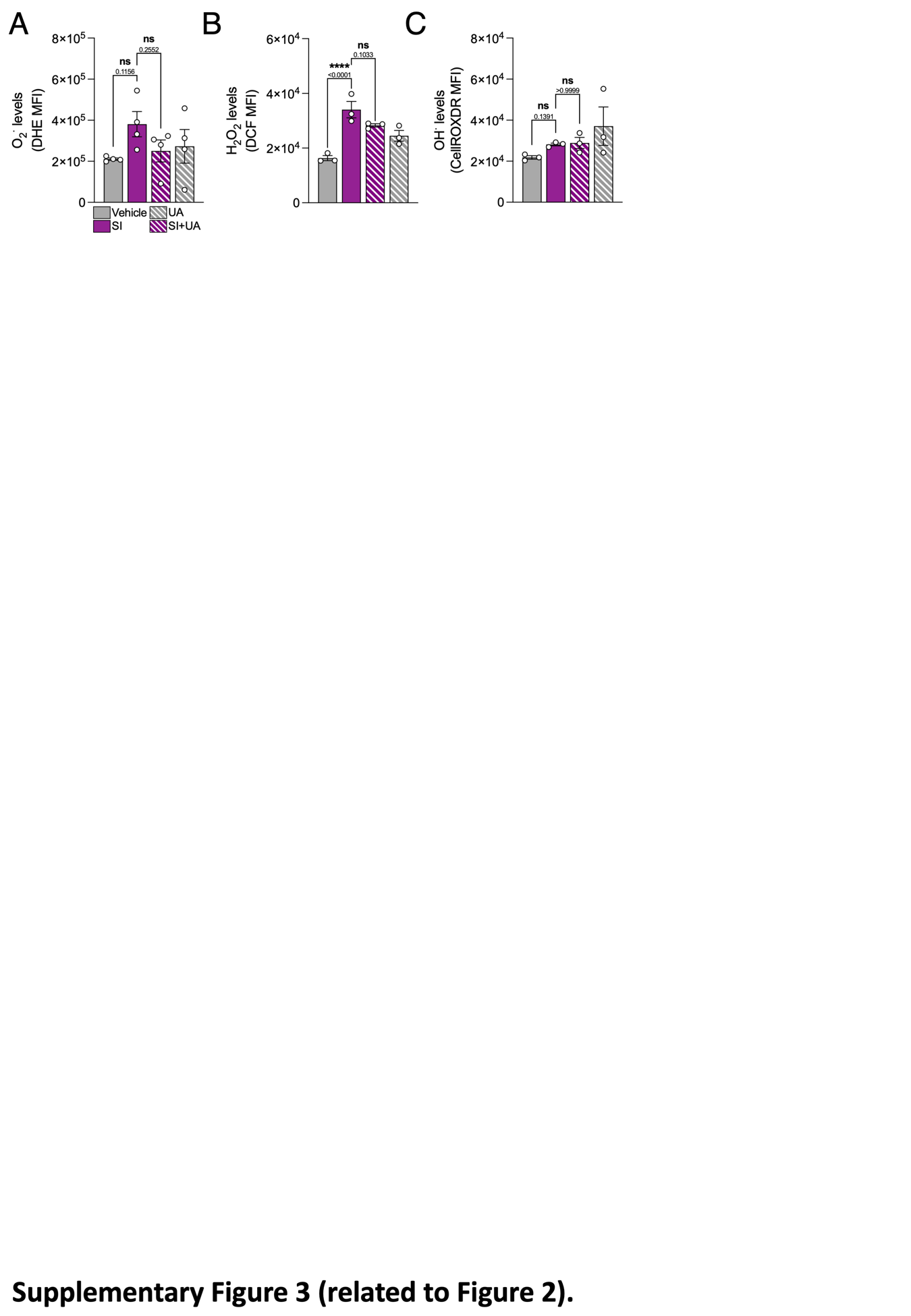
Supplementary Figure 3. UA does not decrease ROS production triggered by SI. A** Quantification of superoxide production (DHE mean fluorescence intensity (MFI)) in ARPE-19 cells treated with 20 mM SI and/or 100 μM UA for 24 hours by flow cytometry. **B** Quantification of hydrogen peroxide production (DCF MFI) in ARPE-19 cells by flow cytometry. **C** Quantification of hydroxyl radical (CellROX Deep Red MFI) in ARPE-19 cells by flow cytometry. All data are expressed as the mean ± s.e.m. Dots represent independent experiments. *P* values were calculated using 1-way ANOVA with Šídák’s *post-hoc* test. *****P*<0.0001, ns: not significant.

**
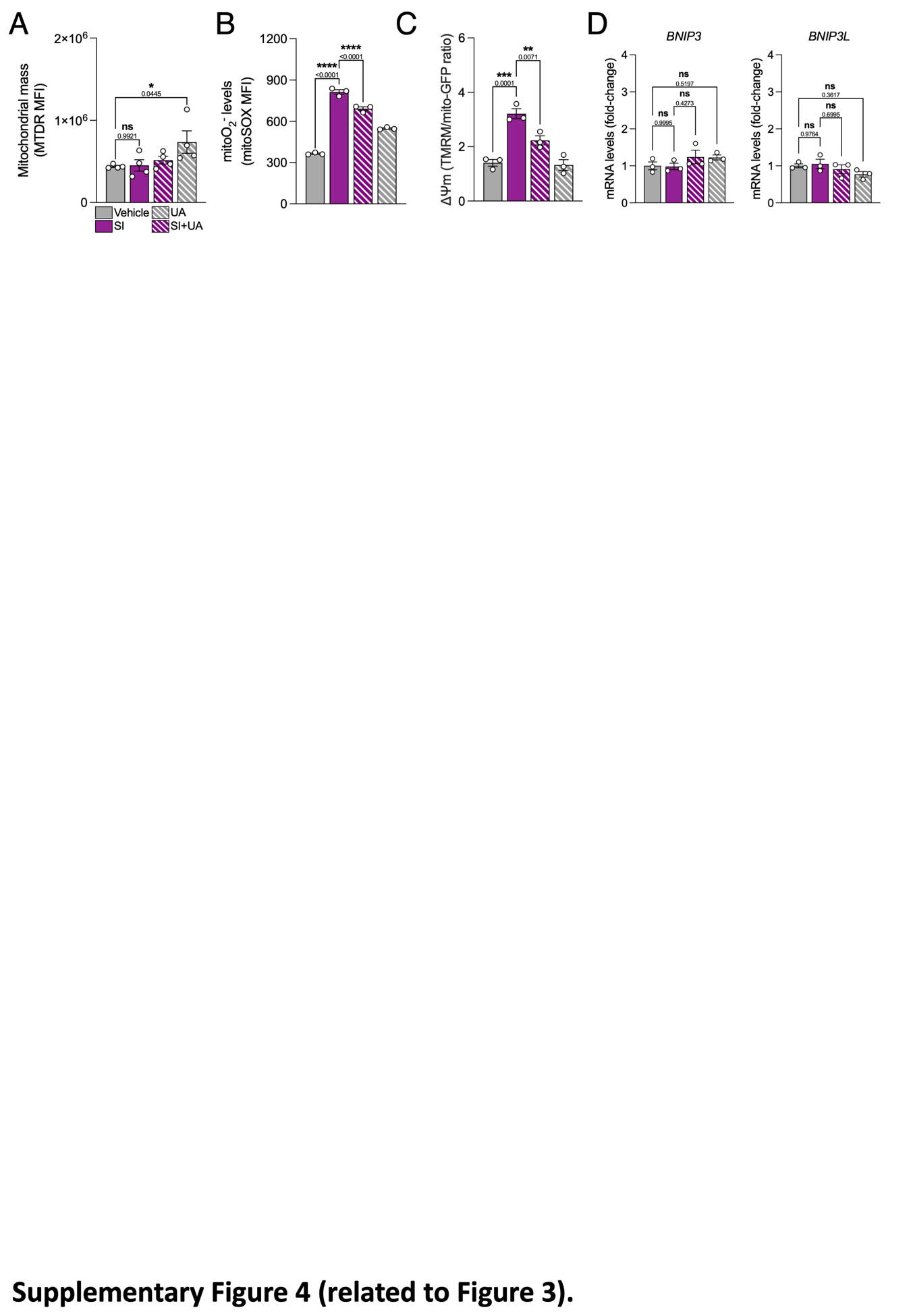
Supplementary Figure 4. UA alleviates signs of mitochondrial dysfunction. A** Quantification of mitochondrial mass (MitoTracker Deep Red MFI) in ARPE-19 cells by flow cytometry. **B** Quantification of mitochondrial superoxide production (MitoSOX Red MFI) in ARPE-19 cells by flow cytometry. **B** Quantification of mitochondrial membrane potential (ΔΨm; TMRM MFI normalized to mitochondrial mass (mito-GFP MFI)) in ARPE-19 cells by flow cytometry. **D** Expression levels of *BNIP3* and *BNIP3L* mRNA in ARPE-19 cells, evaluated by RT-qPCR. All data are expressed as the mean ± s.e.m. Dots represent independent experiments. *P* values were calculated using 1-way ANOVA with Šídák’s *post-hoc* test. *****P*<0.0001, ****P*<0.001, ***P*<0.01, **P*<0.05, ns: not significant.

**
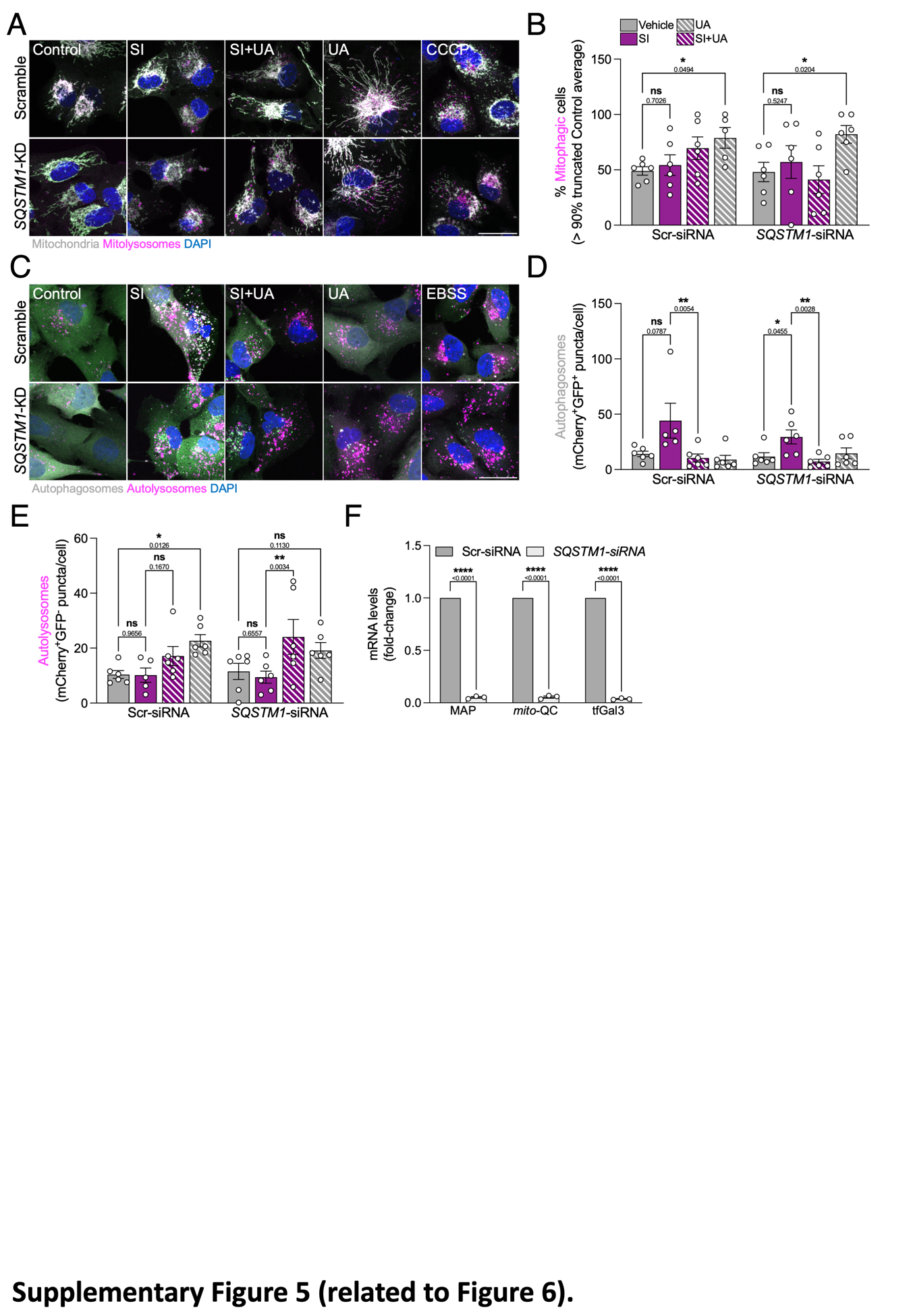
Supplementary Figure 5. p62 is not required for induction of mitophagy and macroautophagy by UA. A** Representative images of ARPE-19 cells transfected with *Scramble* non-targeting siRNA or *SQSTM1*-siRNA and expressing the *mito*-QC reporter, 25 μM CCCP was used as a positive control. **B** Quantification of mitophagy as shown in **A**. **C** Representative images of ARPE-19 cells the *MAP* reporter, EBSS was used as a positive control. **D** Quantification of autophagosome number as shown in **C**. **E** Quantification of autolysosome number as shown in **C**. Knockdown efficiency of *SQSTM1*-siRNA was validated using RT-qPCR. Scale bars, 25. All data are expressed as the mean ± s.e.m. Dots represent biological replicates from three independent experiments. *P* values were calculated using 1-way ANOVA with Fisher’s LSD (**B**, **D**, **E**) *post-hoc* test or unpaired Student’s *t*-test (**F**). *****P*<0.0001, ***P*<0.01, **P*<0.05, ns: not significant.

**Supplementary Tables**

| **Antigen specificity** | **Species** | **Dilution** | **Application** | **Supplier** | **Reference** |
| --- | --- | --- | --- | --- | --- |
| 4-hydroxynonenal | Rabbit | 1:100 | IF | Abcam | ab46545 |
| GFAP | Rabbit | 1:500 | IF | Dako | Z0334 |
| Iba1 | Rabbit | 1:100 | IF | Wako | 019-19741 |
| LC3 | Rabbit | 1:100 | IF | Novus | NB100-2220 |
| SQSTM1/p62 (Human) | Mouse | 1:100 | IF | Abcam | ab56416 |
| POU4F1/Brn3a | Mouse | 1:100 | IF | Millipore | MAB1585 |
| LAMP2 | Mouse | 1:100 | IF | DSHB | H4B4 |
| SQSTM1/p62 (Mouse) | Guinea Pig | 1:100 | IF | Enzo | BML-PW9860 |
| PKCα | Rabbit | 1:500 | IF | Sigma | P4334 |
| ConeArrestin | Rabbit | 1:500 | IF | Millipore | AB15282 |
| VisualArrestin | Mouse | 1:200 | IF | Santa Cruz | sc-166383 |
| F-Actin (Phalloidin) | - | 1:500 | IF | Molecular Probes | A-12380 |
| Alexa-488 Goat anti-Mouse | - | 1:200 | IF | Molecular Probes | A-11001 |
| DyLight-488 Horse anti-Rabbit | - | 1:200 | IF | Vector Laboratories | DI-1088-1.5 |
| Alexa-568 Goat anti-Rabbit | - | 1:200 | IF | Molecular Probes | A-11011 |
| Alexa-568 Goat anti-Mouse | - | 1:200 | IF | Molecular Probes | A-11004 |
| Alexa-568 Goat anti-Guinea Pig | - | 1:200 | IF | Molecular Probes | A-11075 |
| Alexa-647 Goat anti-Rabbit | - | 1:200 | IF | Molecular Probes | A-21244 |
| Alexa-647 Goat anti-Mouse | - | 1:200 | IF | Molecular Probes | A-21235 |

**Supplementary Table 1. Antibodies and dilutions.** IF: immunofluorescence.

| **Name** | **FP-tag** | **Application** | **Supplier** | **Reference** |
| --- | --- | --- | --- | --- |
| MAP | mCherry-GFP | Autophagy reporter | Prof. Ian G. Ganley | - |
| *mito-*­QC | mCherry-GFP | Mitophagy reporter | Prof. Ian G. Ganley | - |
| tfGal3 | RFP-GFP | Lysophagy reporter | Prof, Tamotsu Yoshimori (Addgene) | #64149 |
| mito-GFP | - | Mitochondrial mass | Dr. Aviva Tolkovsky | - |
| PINK1-YFP | - | - | Addgene | #101874 |
| Parkin-EGFP | - | - | Addgene | #45875 |
| TFEB-EGFP | - | - | Addgene | #38119 |

**Supplementary Table 2. Plasmids.** FP: fluorescent protein
